# Downregulation of CDH11 Promotes Metastasis and Resistance to Paclitaxel in Gastric Cancer Cells

**DOI:** 10.7150/jca.48193

**Published:** 2021-01-01

**Authors:** Zhongyin Yang, Chao Yan, Zhenjia Yu, Changyu He, Jianfang Li, Chen Li, Min Yan, Bingya Liu, Yingli Wu, Zhenggang Zhu

**Affiliations:** 1 Department of General Surgery, Gastrointestinal Surgery, Shanghai Key laboratory of Gastric Neoplasms, Shanghai Institute of Digestive Surgery, Ruijin Hospital, Shanghai Jiao Tong University School of Medicine, Shanghai 200025, China.; 2 Hongqiao International Institute of Medicine, Shanghai Tongren Hospital / Faculty of Basic Medicine, Chemical Biology Division of Shanghai Universities E-Institutes, Key Laboratory of Cell Differentiation and Apoptosis of the Chinese Ministry of Education, Shanghai Jiao Tong University School of Medicine, Shanghai 200025, China.

**Keywords:** Gastric cancer, CDH11, peritoneal metastasis, MKN45P cell, paclitaxel resistance

## Abstract

**Background:** Gastric cancer (GC) with peritoneal metastasis has an extremely poor prognosis. Paclitaxel (PTX) intraperitoneal infusion provides an effective treatment for these patients. However, GC patients with peritoneal metastasis who receiving PTX treatments tend to occur PTX-resistance accompany with more aggressive ascites and metastasis. How does this happen is still unknown. Here, we aimed to explore the mechanisms that mediate PTX-resistance and metastasis in GC with peritoneal metastasis.

**Methods:** Ascites samples were collected before PTX infusion and after the relapse in 3 GC patients. To determine the expression of significantly changed proteins, we performed tandem mass tag (TMT) quantitative proteomics. Immunohistochemistry (IHC) staining and western blot were performed to confirm the expression of CDH11 in the PTX-resistant tissues and MKN45P-PR cells. Invasion and migration of GC cells were examined by* in vitro* transwell and wound healing assays and *in vivo* dissemination experiments.

**Results:** CDH11 expression was downregulated in the relapsed PTX-resistant ascites, tissues and the PTX-resistant cell line MKN45P-PR. Inhibition of CDH11 expression promoted the invasion, migration and PTX resistance of MKN45P cells, while overexpression of CDH11 repressed these biological functions. Moreover, tumors disseminated in the mice peritoneal cavity induced by MKN45P-PR cells and shCDH11 cells displayed higher metastatic ability and resistance to PTX treatment.

**Conclusions:** Our results reveal that CDH11 is inhibited in the relapsed PTX-resistant patients and the downregulated CDH11 expression promotes GC cell invasion, migration and PTX resistance. CDH11 may have the potential to serve as a predictable marker for the occurrence of PTX resistance in GC patients with peritoneal metastasis.

## Introduction

Gastric cancer (GC) is the fifth most common malignancy and the third leading cause of cancer-related deaths worldwide. In China GC is the third leading cause of mortality; approximately 30.0 people per 100 000 developed GC in 2000, and 21.48 people per 100 000 die of this disease 1.

Peritoneal metastasis (PM) is the most common form of metastasis in GC in China. Patients with PM often have malignant ascites, which is associated with low quality of life, intestinal obstruction and an extremely poor prognosis. Numerous strategies have been used to treat PM, including aggressive cytoreductive surgery (CRS) 2, and hyperthermic intraperitoneal chemotherapy (HIPEC) 3-5, but none has provided a satisfactory clinical outcome. Recent studies have demonstrated that the intraperitoneal (IP) administration of paclitaxel (PTX) exhibits positive effects on PM in GC patients 6. When administered intraperitoneally, PTX exhibits delayed clearance from the peritoneal cavity due to its high molecular weight and fat solubility, leading to a high concentration of PTX in the peritoneal cavity 7, 8. In a phase Ⅱ study, Ishigami *et al*. demonstrated that combination treatment with intravenous and intraperitoneal infusion of PTX is well tolerated and that the 1- year overall survival rate reaches 78% in GC patients with PM 9. Further, their phase Ⅲ clinical trial suggests possible clinical benefits of IP paclitaxel for GC, with a 3-year overall survival rate of 21.9% in the IP group 10. Generally, a larger volume of ascites is associated with the severity of PM and poorer survival, especially for those with recurrent ascites. However, there are few studies about the recurrence of ascites and the mechanism is still elusive.

Based on these results, we launched our phase Ⅱ and phase Ⅲ clinical trials for patients with PM. In the trial, some patients showed a better response to PTX with IP infusion, and malignant ascites were quickly controlled after 1-3 cycles of chemotherapy. However, in some patients, the ascites recurred after several cycles of the therapies and showed resistance to PTX therapy, and displayed more aggressive metastasis. Based on these clinical findings, we selected ascites samples from 3 patients before PTX infusion and after the relapse of malignant ascites. Then, the samples were analyzed by TMT quantitative proteomics, and 19 differentially expressed proteins were identified. Through analysis we found that the protein CDH11 was closely correlated with the relapse of the disease.

In tumors, CDH11 was reported to exert complicated functions 11, 12. Nakajima *et al*. showed that CDH11 overexpression had a suppressive effect on pulmonary metastasis of osteosarcoma 13. Particularly, in head and neck cancer cells, CDH11 inhibited proliferation and invasion 14. In this study, CDH11 expression was verified in one PTX-resistant cell line, MKN45P-PR. Overexpression of CDH11 resulted in reduced resistance to PTX, while downregulation of CDH11 led to resistance to PTX. Furthermore, downregulation of CDH11 promoted the invasion and migration of GC cells, while overexpression of CDH11 repressed these biological functions. These results support the view that downregulation of CDH11 promotes the resistance of PTX and metastasis in GC cell line MKN45P, which has a high potential for peritoneal dissemination.

## Materials and Methods

### Patients selection and samples collection

The selected patients met the following criteria: 1) GC patients with peritoneal metastasis but no other metastasis; 2) patients received no other treatments before the infusion of PTX; and 3) the malignant ascites disappeared after the 1-3 cycles of PTX chemotherapy but relapsed later with more metastasis and were resistant to PTX treatment. Three patients who received PTX chemotherapy between January 2018 and October 2018 and met all the above criteria were selected (Supplementary Table S1).

Ascites samples from the 3 patients were collected from the peritoneal cavity during the 1st and 2nd laparoscopic exploration. Fifty milliliters of ascites from patients was collected, transferred into 50 ml tubes and centrifuged at 2000 x g for 15 min at 4 °C to remove cell debris. The supernatant was then stored at - 80 °C until further use. Gastric cancer tissues and ovarian specimens were collected from GC patients with peritoneal metastasis who underwent chemotherapy between December 2017 and December 2018 in the Department of Surgery, Ruijin Hospital, Shanghai Jiaotong University School of Medicine. Gastric cancer and PTX-resistant GC (GC-PR) tissues were obtained through esophagogastroscopy before PTX chemotherapy and after the occurrence of PTX resistance. Ovarian specimens were obtained by laparoscopic surgery before PTX chemotherapy and after the occurrence of PTX resistance. After being fixed with formalin and embedded in paraffin, all samples were cut into consecutive slides and immunohistochemistry (IHC) were then performed. Samples were collected at Ruijin Hospital Shanghai Jiao Tong University under informed consent and internal review board approval.

### Ascites management, quality control and LC-MS/MS

Moderate SDT-lysis buffer was added to the ascites samples, and the ratio was 1:10 sample to buffer and boiled at 95 °C for 5 min. Before sample processing, the lysate was clarified by centrifugation at 14,000 x g for 15 min. The supernatant was collected and quantified by the bicinchoninic acid (BCA) assay 15, and the quality and quantity of the protein was determined for the next step. Twenty micrograms of each extracted protein was analyzed by SDS-PAGE, and Coomassie blue staining was performed. After enzymolysis, peptide labeling was performed following TMT Multiplex manufacturer's instructions (Thermo Fisher Scientific, Bremen, Germany) with a unique isobaric label (TMT Sixplex™ Isobaric Label Reagent Set). The samples were separated by Easy nLC chromatography and submitted to analysis using high-resolution Q Exactive hybrid quadrupole-Orbitrap LC-MS/MS (Thermo Fisher Scientific, Bremen, Germany).

**Figure 1:**
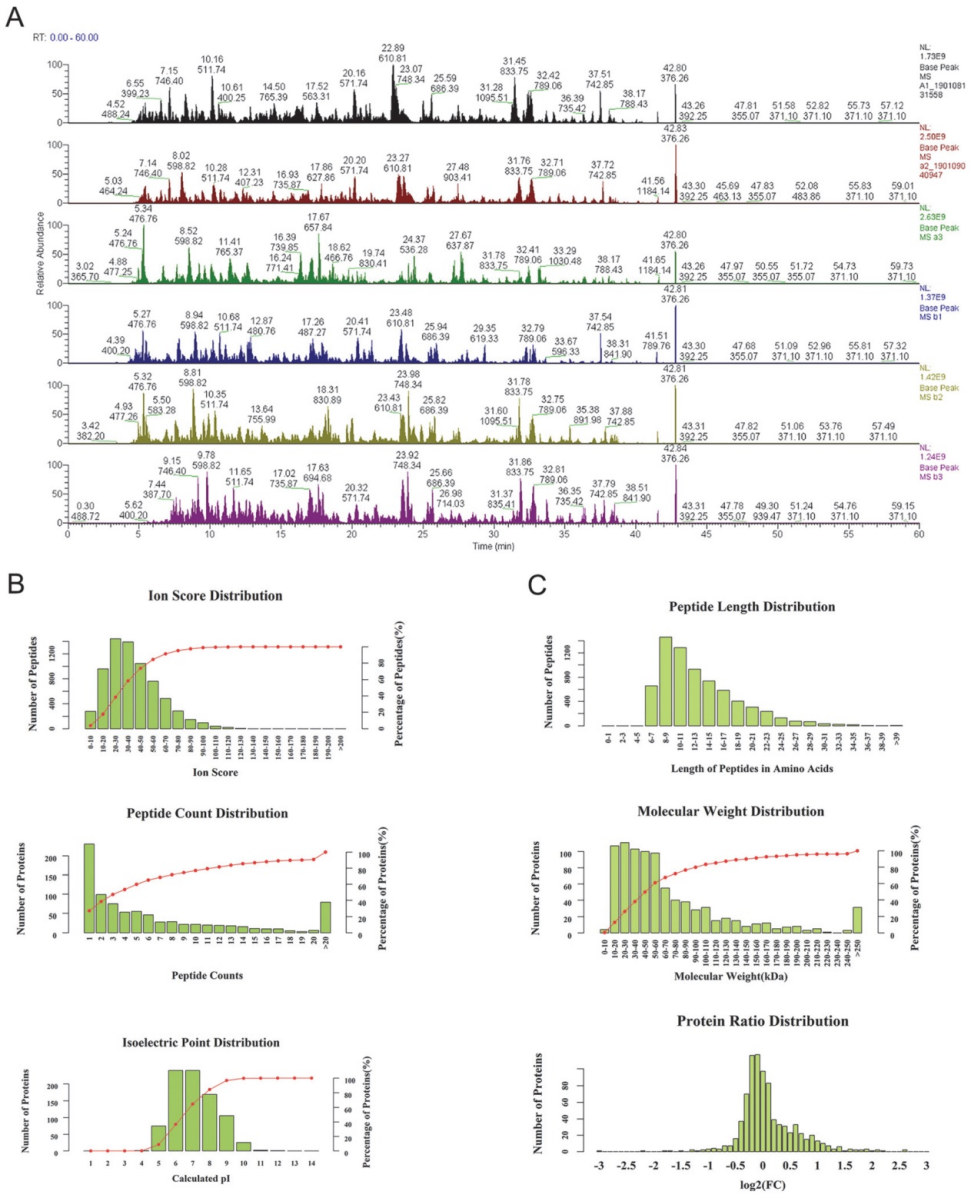
The quality control of peptides after enzymolysis of the ascites proteins. (A) The base peak chromatogram of all 6 patients ascites samples. a1-a3: before PTX treatment, b1-b3: after the relapse and PTX resistance. (B) The authentication and quantitation evaluation of the MS by Iron score distribution, Peptide count distribution, Isoelectric point distribution and (C) Peptide lenth distribution, Molecular weight distribution and Protein ratio distribution.

As the base peak intensity chromatograms of LC-MS/MS analysis indicated, the consistence among all protein groups was good (Figure 1). The mass deviation of more than 90% peptides of all identified peptides was within 8 ppm, which indicates that the MS instrument is in good condition and that the data are believable (Supplementary Figure 1). Furthermore, the peptides ion score, count, isoelectric point, length, molecular weight, and protein ratio distribution were determined for peptides quality control (Figure 1B, C). Mascot 2.6 and Proteome Discoverer 2.1 software was used for comparison with the protein library and the number of authenticated proteins.

### Data processing

RAW files were processed by using Proteome Discoverer (PD version 2.1; Thermo Fisher Scientific, Bremen, Germany) with the Mascot algorithm. The data processing was performed as previously described 16. The top 3 peptides were used for area calculation. For protein grouping, the strict parsimony principle was applied. For peptide and protein quantifiers, normalization mode was set as the total peptide amount, and scaling mode was set as the channels average. Proteins that conformed to *P* < 0.05 (Student's *t* test) were deemed differentially expressed. Hierarchical clustering analysis and TreeView analysis were performed to generate a dendrogram for each cluster of genes based on their expression profiling similarities.

### Establishment of a paclitaxel‑resistant MKN45P-PR cell line

The human gastric cancer cell line MKN45P, a GC cell line with high potential for peritoneal dissemination, was kindly provided by Professor Joji Kitayama (Jichi Medical University, Tochigi, Japan) 17. The cells were cultured in RPMI 1640 (Gibco, Waltham, MA, USA) supplemented with 10% fetal bovine serum (FBS; Gibco) and incubated at 37 ˚C in a humidified atmosphere of 5% CO_2_. Paclitaxel‑resistant MKN45P-PR cells were established by continuous exposure to stepwise‑increasing concentrations of paclitaxel (PTX) (Selleck Chem, Houston, TX, USA). MKN45P cells were initially cultured in medium containing a low concentration of PTX (1 nM). Then the cells were cultured in medium with concentration gradient of PTX (2 nM, 4 nM, 5 nM, 6 nM, 8 nM, 10 nM, 12 nM, 14 nM, 16 nM, 18 nM, and 20 nM). The cells were cultured in each concentration of PTX for 2 weeks. Finally, the cells that were cultured and survived in medium with a high concentration of PTX (20 nM) were defined as PTX‑resistant MKN45P-PR cells 18.

### DNA/shRNA transfection

pECMV-CDH11-3×FLAG plasmid DNA was purchased from Miaoling Plasmid Platform (Hubei, China). Human CDH11 cDNA were amplified from gastric cancer cells by PCR (forward primer: 5'-ATGAAGGAGAACTACTGTTTAC-3' and reverse primer: 5'- TTAAGAATCGTCATCAAAAGTG -3'), and then the PCR products were purified with DNA extraction kits. The PCR products and pECMV-3×FLAG vector were digested with *HindIII* and *BamHI* at 37 ℃ for 4 h, and purified. The purified DNA was ligated with pECMV-3×FLAG by using T4 DNA ligase at 16 C for 12 h to generate the pECMV-CDH11-3×FLAG constructs for transfection. To knockdown the expression of CDH11, short RNA targeting sequences (shCDH11 #1, 5'-GGGAAATTGTTTATGTGTT-3'; shCDH11 #2, 5'- GCCAAGTTAGTGTACAGTA-3') were synthesized, annealed and ligated into the lentivirus pGIPZ vector. Lentivirus shCDH11s were prepared in HEK293T cells packaged by pMD2G and pSPAX2. The MKN45P cells were incubated in medium containing virus particles and supplemented with 8 μg/ml polybrene for 6 h, followed by replacement of fresh medium. The cells were selected with 2 μg/ml puromycin starting after 48 h after virus infection for a duration of 7 -10 days, and the stable cells were collected and expanded for next step experiments.

### Invasion and migration assays

Experiments were conducted using 24-well plates and 8 μm transwell inserts (Corning, NY, USA). MKN45P or MKN45P-PR cells suspensions (1×10^5^ each) were seeded in the chambers for invasion or migration assays. For migration assays, tumor cell suspended in 500 μl fetal bovine serum (FBS) free medium and cultured in the upper chamber. FBS-conditioned medium (10%) (750 μl) was added to the bottom chamber and the cells were cultured for 48 h. For invasion assay, chambers with Matrigel-coated inserts were used. After 48 h of culture, the cells attached to the undersurface of the membrane were fixed in methanol for 30 min and the cells on the upper surface of the filter were removed and stained with crystal violet for 15 min. The number of cells that migrated to the lower side of the inserts and invaded through the Matrigel layer was counted in five random fields with an Olympus BX51 microscope.

### Wound healing assay

A total of 3×10^5^ cells were seeded in 6-well plates, when the cells reached 80% to 90% confluence, the cell layer was scratched with a pipette tip. The cells were washed three times followed by treatment with FBS-free medium. The wound closure was monitored at 48 h and 72 h by comparing the wound distance ratio at 0 h. At different time points, images of the plates were acquired using a microscope. The experiment was independently repeated three times.

### *In vivo* dissemination experiments

Animal experiments were performed using 5-week-old male BALB/C nude mice in accordance with the guidelines and approval of the Institutional Animal Care and Use Committee of Ruijin Hospital, Shanghai Jiao Tong University. Mice were intraperitoneally injected with 2×10^6^ cells (MKN45P, MKN45P-PR, or MKN45P/shCDH11) suspended with PBS (five mice per group). Two weeks after the injection of tumor cells, vehicle or PTX 20mg/kg injected intraperitoneally for 14 days Mice were euthanized after 4 weeks after injection, and the quantity of peritoneal nodules in the abdominal cavity was calculated.

### Protein Quantification and Western Blot Analyses

Total cell pellets were lysed in 1×SDS-PAGE lysis buffer and boiled as described previously. A total of 20 μg of protein was loaded onto a 10% sodium dodecyl sulfate polyacrylamide gel and then transferred to nitrocellulose membranes. Membranes were blocked and then incubated with primary antibodies overnight at 4 ℃. The primary antibodies recognized CDH11 (4442S, Cell Signaling Technology), E-cadherin (14472, Cell Signaling Technology), and the β-actin antibody (PM053-7, MBL) was used as a loading control. On the following day, membranes were washed with 1×tris-buffered saline + Tween (TBST), incubated with secondary antibodies conjugated to horseradish peroxidase (1:1,000) for 1 h, and washed 3 times with 1×TBST. The quantitative changes in the luminescence were estimated with LAS1000 UV mini and Multi Gauge Ver. 3.0 (Fuji Film, Tokyo, Japan).

### IHC staining

IHC staining was performed in paraffin-embedded tissues according to a previous study 19. The following primary antibodies were used: rabbit anti-CDH11 (1:400) followed by an incubation with a biotinylated secondary antibody. The staining intensity was graded in four segments on a 3-point scale (staining scores): no staining (0 points), light brown staining (1 point), brown staining (2 points) and dark brown staining (3 points).

### Statistical Analyses

Experimental results were performed with Graphpad prism 8.0. The results were appeared as mean ± SD. The paired *t-*test was used for statistical analysis between two groups. *P* < 0.05 was considered significant.

## Results

### Identification of differentially expressed proteins

**Figure 2:**
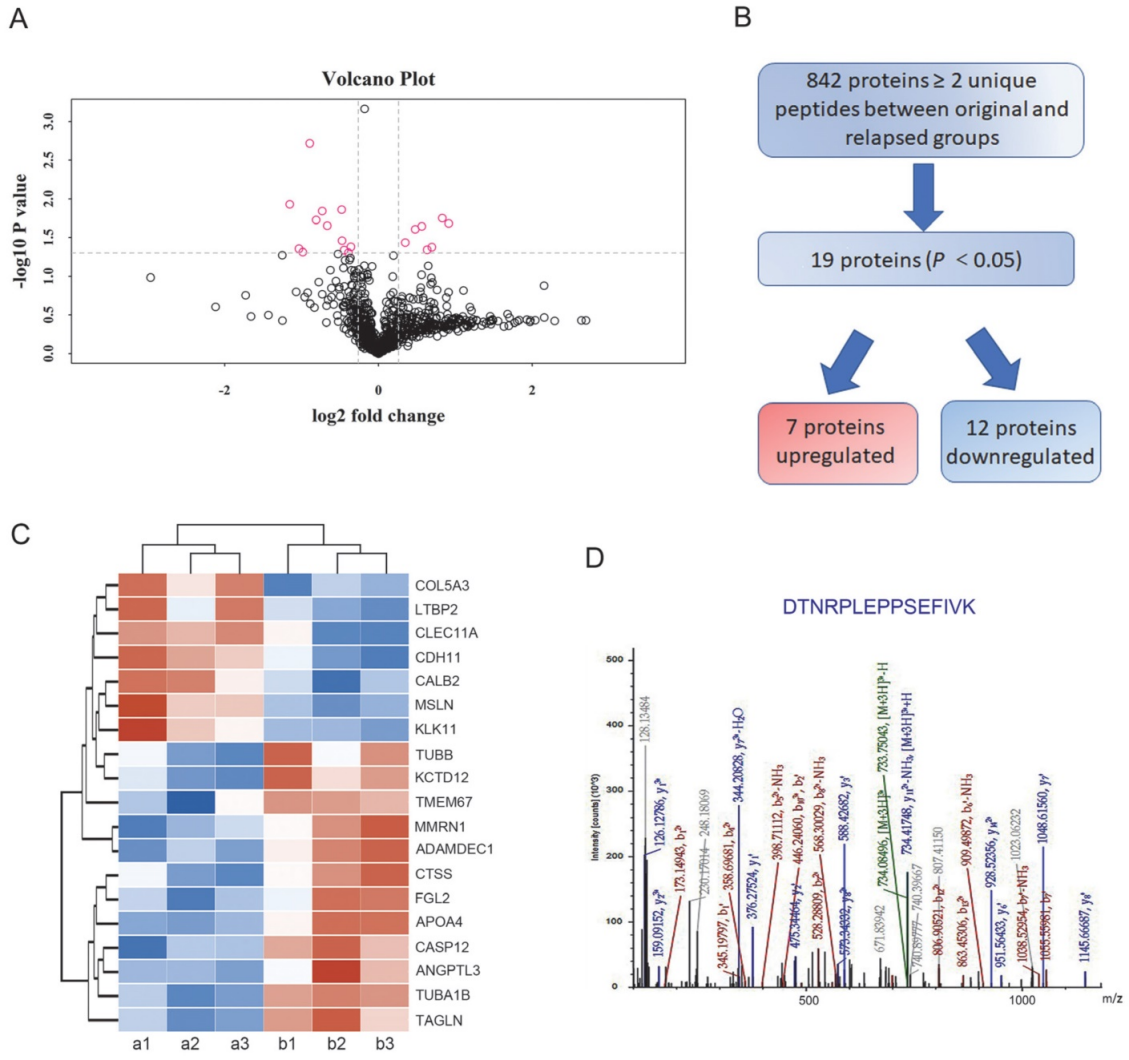
Differentially expressed proteins between the original and relapsed ascites. (A) Volcano plots demonstrated differential proteins expression in the relapsed groups compared to the untreated original groups, plotted against -log_10_ (P) in the y axis and log_2_ (fold) in the x axis. (B) Flow diagram for the analysis of differentially expressed proteins between the original and relapsed ascites. (C) Clustering analysis of 19 differentially expressed proteins. (D) Fragmentation spectrum of the identified CDH11 peptide.

In total, 842 proteins identified by TMT-based proteomics were represented by ≥ 2 unique peptides (Supplementary Table S2). When these data plotted against -log_10_ (P) in the y axis, volcano plots demonstrated differential expression in the relapsed groups compared to the untreated original groups (Figure 2A). Among the proteins, 454 proteins were identified as upregulated and 388 proteins were downregulated in the treated ascites samples compared to the untreated control samples. Proteins showing *P*<0.05 were identified as significantly differentially expressed proteins. Following these criteria, 7 upregulated and 12 downregulated proteins were determined to be significantly regulated (Figure 2B). Furthermore, highly regulated protein classes in both upregulated and downregulated proteins were extracted for cluster analysis (Figure 2C). In these dysregulated proteins only CDH11 and Tubulin alpha-1B (TUBA1B) were reported to play a role both in metastasis and PTX resistance 20, 21. Based on preliminary experimental results, the metastasis associated cadherin protein CDH11 was selected for further study (Figure 2D).

### CDH11 is expressed at low levels in PTX-resistant cell line and tissues

**Figure 3:**
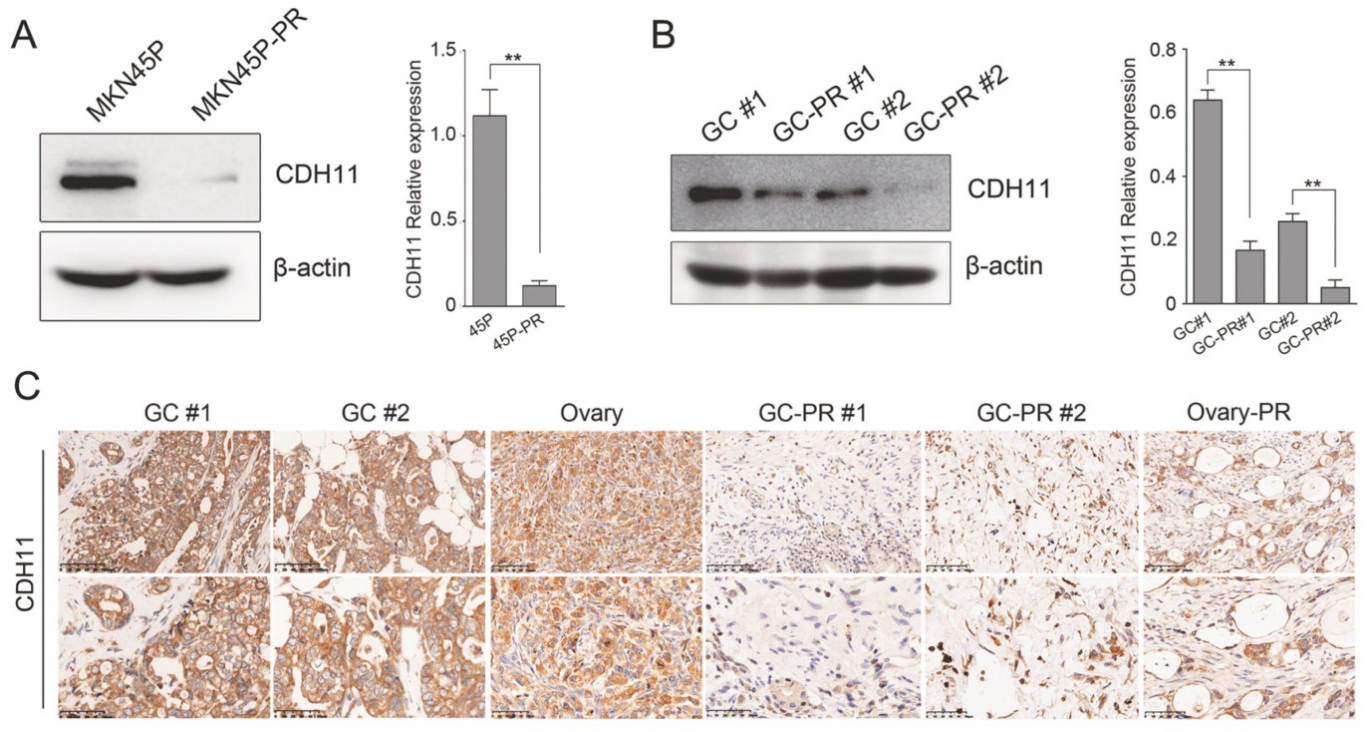
CDH11 is lowly expressed in PTX-resistant cell line and tissues. (A) CDH11 expression was examined by western blot in MKN45P and MKN45P-PR cells. (right) The relative expression of the CDH11 protein levels in the cell lines. The histogram indicates the signal intensity of the proteins against β-actin (mean ± SD, **P < 0.01). (B) Western blot analysis for the expression of CDH11 in 2 pairs of human GC samples. (right) The relative expression of the CDH11 protein levels in tissues. The histogram indicates the signal intensity of the proteins against β-actin (mean ± SD, **P < 0.01). (C) Representative IHC staining images of CDH11 in GC tissues (GC #1 staining intensity: 3 points; GC #2 staining intensity: 3 points), metastatic tissues (Ovary staining intensity: 2 points) and PTX-resistant tissues (GC-PR #1, GC-PR #2 and Ovary-PR staining intensity: 1 point; up: ×200; below: ×400).

The expression of CDH11 was verified in MKN45P and PTX-resistant cell line MNK45P-PR. CDH11 expression was observed significantly downregulated in MKN45P-PR cells compared to MKN45P cells (Figure 3A). The findings were consistent with the results in two GC tissues and PTX-resistant GC tissues (Figure 3B). To further illuminate the expression pattern of CDH11, we performed IHC in GC tissues, metastatic ovarian tissue, PTX-resistant GC tissues and metastatic PTX-resistant ovarian tissues. The results showed that the PTX-resistant tissues displayed a reduced expression of CDH11 (Figure 3C). Therefore, CDH11 expression is downregulated in PTX-resistant cell line MNK45P-PR, PTX-resistant GC tissues and metastatic PTX-resistant tissues.

### CDH11 downregulation promotes MKN45P cell invasion, migration and PTX resistance *in intro* and *in vivo*

**Figure 4:**
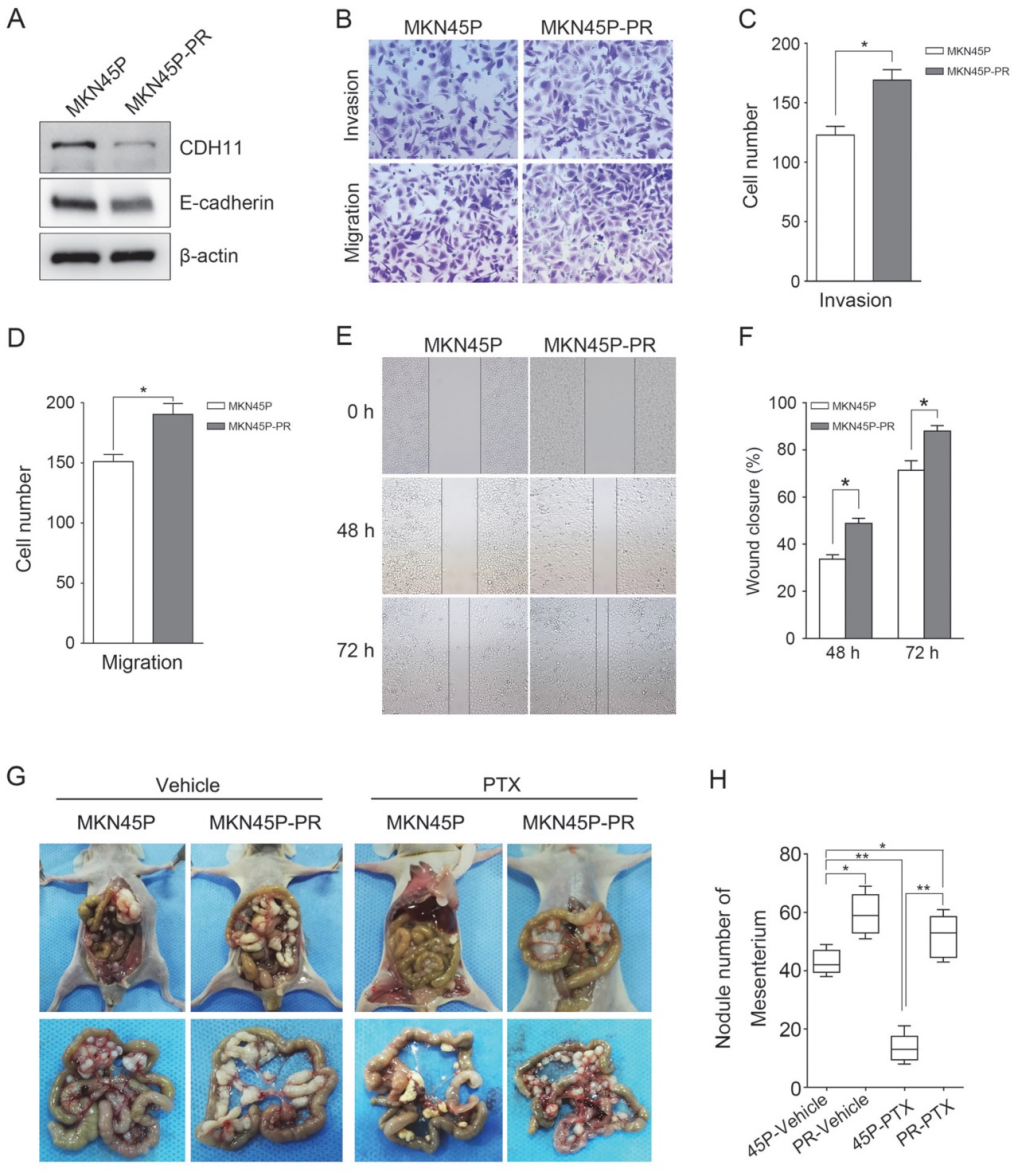
MKN45P-PR cells demonstrates metastatic and PTX-resistant characteristics. (A) CDH11 and E-cadherin expression were examined by western blot. (B-D) A matrigel invasion and migration assay and a scratch-wound assay (E, F) were performed. The wound closure (%) was calculated (mean ± SD, *P < 0.05). (G) MKN45P and MKN45P-PR cells were intraperitoneally injected into the nude mice, and treated with vehicle and PTX; macroscopic images of the peritoneal disseminations are shown, and the number of peritoneal nodules from each mouse were measured (H) (mean ± SD, *P < 0.05).

Furthermore, to test whether inhibition of CDH11 influences the invasion and migration of GC cells, we first examined the expression of E-cadherin in MKN45P-PR cells. As expected, the expression of CDH11 and E-cadherin was suppressed in MKN45P-PR cells (Figure 4A). Transwell and wound healing assays also demonstrated that MKN45P-PR cells had an enhanced metastatic ability (Figure 4B-F).

We further evaluated whether inhibition of CDH11 could promote metastasis and resistance to PTX *in vivo*. MKN45P and MKN45P-PR cells were intraperitoneally injected into mice. Vehicle and PTX were intraperitoneally injected continuously for 14 days. The average number of tumor nodules in the abdominal cavity of the mice injected with MKN45P-PR cells was substantially increased compared to that of the mice injected with MKN45P cells. The number of tumor nodules derived MKN45P was substantially reduced after intraperitoneal injection with PTX; however, in the MKN45P-PR group the number of tumor nodules increased and displayed resistance to PTX treatment (Figure 4G, H).

**Figure 5:**
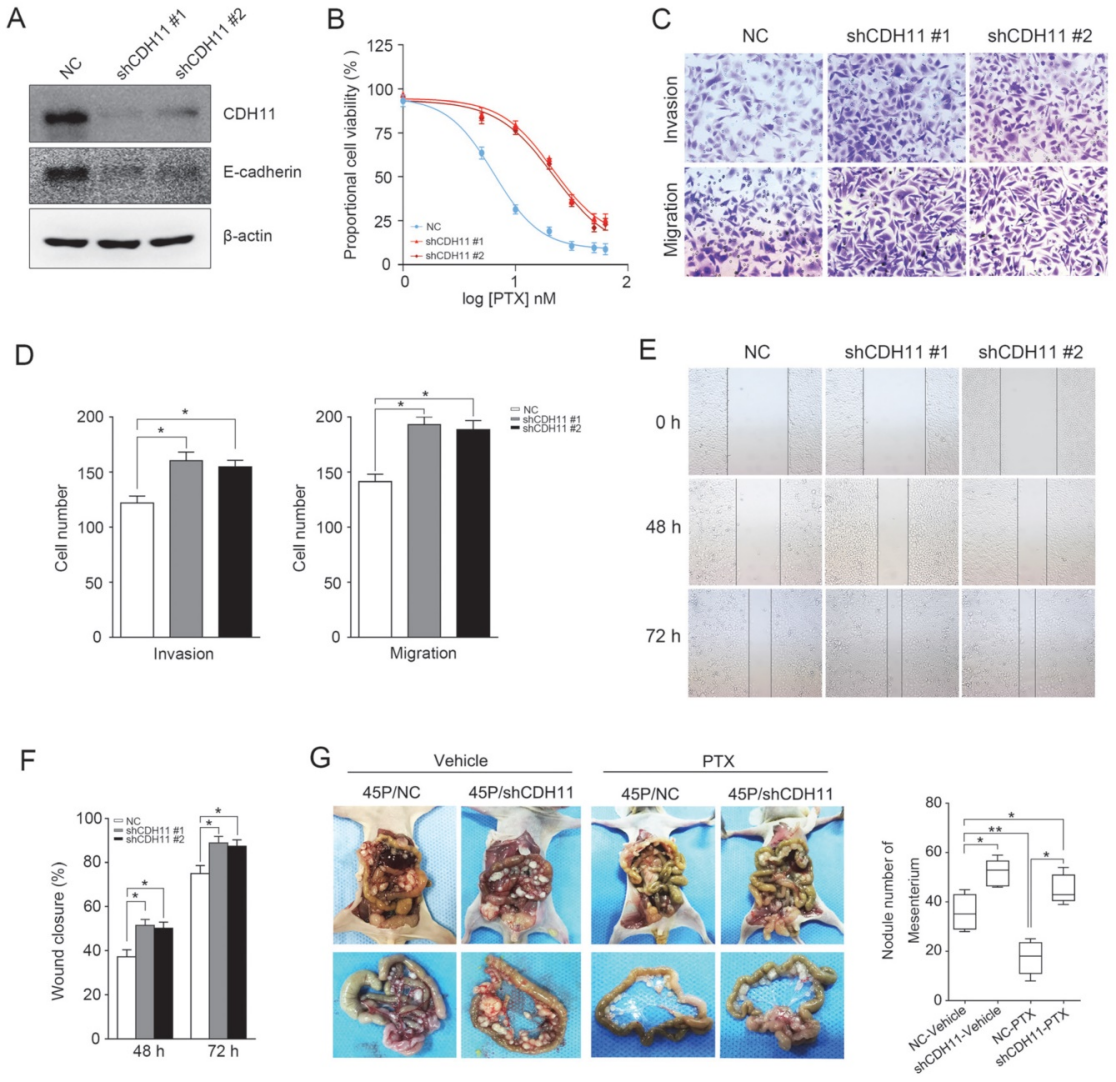
Downregulation of CDH11 expression promotes the invasion, migration and PTX resistance *in vitro* and *in vivo*. (A) Western blot was conducted to examine the expression of CDH11 and E-cadherin after the knockdown of CDH11. (B) CDH11 knockdown MKN45P cells were treated with various concentrations of PTX (1-60 nM), after 48 h, IC_50_ values for PTX were measured by CCK-8 assays. (C, D) A matrigel invasion and migration assay and a scratch-wound assay (E, F) were performed to examine the invasion and migration of CDH11 knockdown cells. The wound closure (%) was calculated (mean ± SD, *P < 0.05). (G) MKN45P/NC and MKN45P/shCDH11 cells were intraperitoneally injected into the nude mice, and treated with vehicle and PTX; macroscopic images of the peritoneal disseminations are shown, and the number of peritoneal nodules from each mouse were measured (right) (mean ± SD, *P < 0.05).

Next, two specific shRNAs were used to downregulate the expression of CDH11 in MKN45P cells. As demonstrated in Figure 5A, CDH11 and E-cadherin expression was obviously inhibited. Importantly, the resistance of MKN45P cells to PTX was increased when CDH11 expression was downregulated (Figure 5B). Furthermore, the invasion and migration of MKN45P cells were increased when CDH11 expression was inhibited, as confirmed by tanswell and wound healing assays (Figure 5C-F). To further observe the effect of downregulation of CDH11 on PTX resistance *in vivo*, we intraperitoneally injected MKN45P/NC and MKN45P/shCDH11 cells into mice, and vehicle and PTX were intraperitoneally injected for 14 days. As shown in Figure 5G, the average number of tumor nodules in the abdominal cavity of the mice injected with the MKN45P/shCDH11 cells was substantially increased compared with that of the mice injected with the MKN45P/NC cells. The number of tumor nodules derived MKN45P/NC were strongly reduced after intraperitoneal administration of PTX; however, in the MKN45P/shCDH11 group the number of tumor nodules increased and displayed resistance to PTX treatment. Collectively, these results indicate that inhibition of CDH11 promotes the metastasis and resistance of MKN45P cells to PTX* in vitro* and *in vivo.*

### Forced expression of CDH11 inhibits PTX resistance, invasion and migration

**Figure 6:**
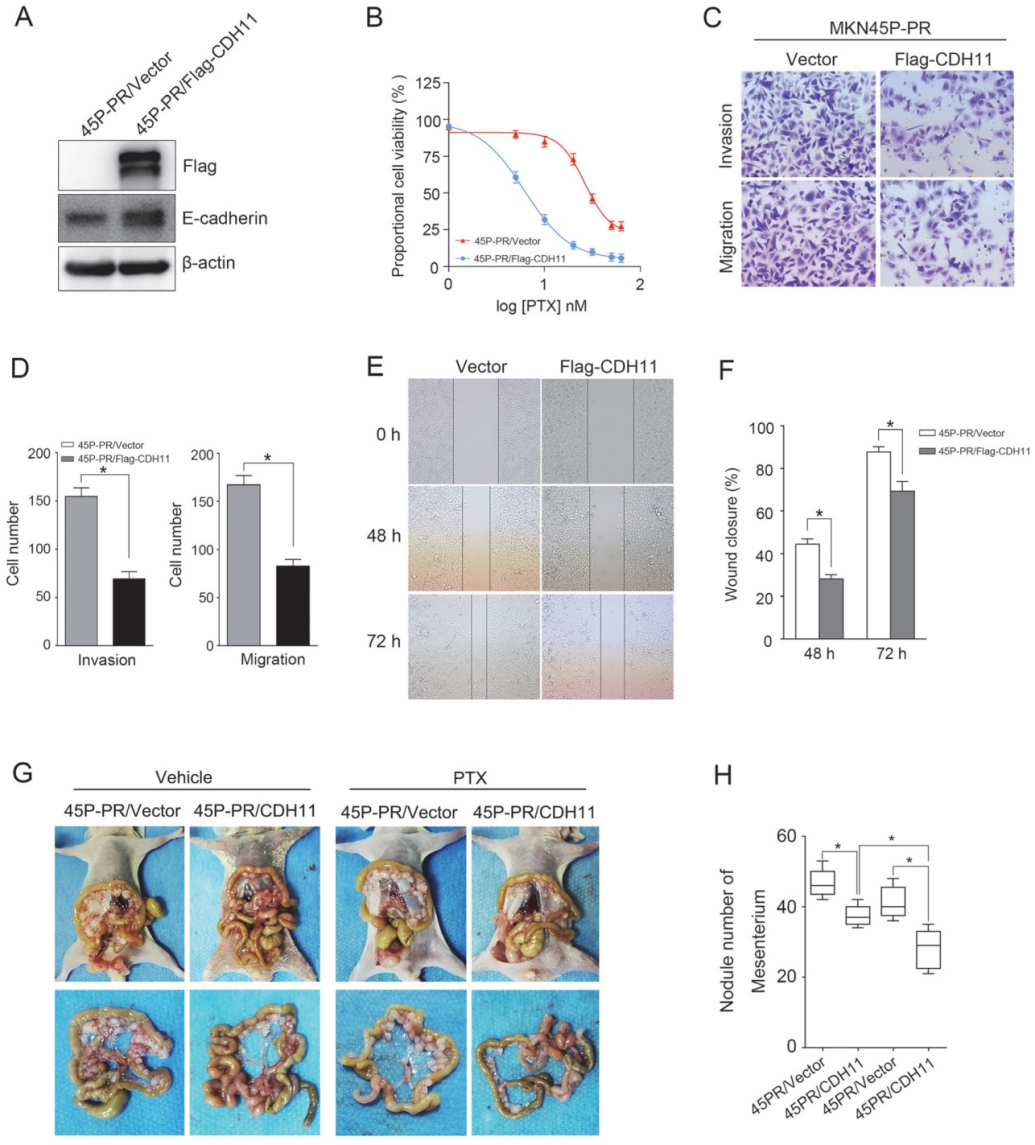
CDH11 overexpression suppresses PTX resistance, invasion and migration of MKN45P-PR cells. (A) Flag-tag and E-cadherin expression were examined by western blot in Flag-CDH11 ectopically expressed cells. (B) MKN45P cells overexpressed with CDH11 were treated with various concentrations of PTX (1-60 nM), after 48 h, IC_50_ values for PTX were measured by CCK-8 assays. (C, D) A matrigel invasion and migration assay and a scratch-wound assay (E, F) were performed to examine the invasion and migration of CDH11 overexpressed cells. The wound closure (%) was calculated (mean ± SD, *P < 0.05). (G) MKN45P-PR/Vector and MKN45P-PR/Flag-CDH11 cells were intraperitoneally injected into the nude mice, and treated with vehicle and PTX; macroscopic images of the peritoneal disseminations are shown, and the number of peritoneal nodules from each mouse were measured (right) (mean ± SD, *P < 0.05).

To determine the effect of CDH11 expression on the PTX resistance and metastasis, we ectopically expressed Flag-tagged CDH11 in MKN45P-PR cells. The overexpression of CDH11 increased the expression of E-cadherin (Figure 6A). Additionally, the overexpression of CDH11 impaired the cell resistance to PTX treatment (Figure 6B). Moreover, the invasion and migration of MKN45P-PR cells were decreased, as indicated by transwell and wound healing assays (Figure 6C-F).

To observe the effect of CDH11 overexpression on the metastasis and PTX resistance *in vivo*, we intraperitoneally injected MKN45P-PR/Vector and MKN45P-PR/Flag-CDH11 cells into mice. As showed in Figure 6G and H, the average number of tumor nodules in the abdominal cavity of the mice injected with the MKN45P-PR/Flag-CDH11 cells was obviously decreased compared with that of the mice injected with the MKN45P-PR/Vector cells. Importantly, the number of tumor nodules derived MKN45P-PR/Flag-CDH11 cells were substantially reduced after intraperitoneal injection with PTX. Taken together, these results indicated that CDH11 overexpression repressed the PTX resistance, invasion and migration of MKN45P-PR cells.

## Discussion

The treatment of advanced GC, especially in patients with PM is a major challenge worldwide. Intraperitoneal chemotherapy with PTX provides a promising regimen for these patients. However, the likelihood of resistance is a disadvantage of PTX. In the current study, we are the first to use TMT technology to screen for potential biomarkers in recurrent ascites with metastasis. The TMT approach enabled the detection of proteins that were dysregulated before and after the occurrence of PTX resistance. With bioinformatic analysis, 7 upregulated and 12 downregulated proteins were determined to be significantly regulated, and only the CDH11 and TUBA1B proteins were associated with metastasis and PTX resistance. CDH11 was further confirmed to be downregulated and mediated the invasion and migration of MKN45P cells. *In vitro* experiments showed that CDH11 expression was reduced in the PTX-resistant cancer tissues and a PTX-resistant gastric cancer cell line MKN45P-PR. The inhibition of CDH11 obviously promoted the invasion, migration and PTX resistance of MKN45P cells, whereas forced expression of CDH11 inhibited these biological abilities. Notably, in the IP dissemination mouse model, downregulation of CDH11 was required for the metastasis and acquisition of PTX resistance.

CDH11 is a mesenchymal cadherin that is frequently expressed in many cancers and is associated with aggressive cancer behaviors, such as invasion and migration. For instance, CDH11 promotes metastases to the bone in breast cancer, due to the high homologous binding affinity of cancer cells for the strongly CDH11-expressing osteoblasts 22. Additionally, CDH11 can promote small GTPase Rac activity by facilitating the plasma translocation of the Rac-specific GEF Trio, which promotes breast cancer cell migration 23. However, CDH11 was also reported to suppress metastasis in certain cancers. In head and neck and melanoma, loss of CDH11 expression enhanced the metastatic phenotype 24. Interestingly, it has been reported that there could be an interplay between PTX resistance and EMT because it is associated with a common pathway involving glycosylation 25. Wu *et al*. found that resistance to PTX was associated with reduced levels of E-cadherin and increased cell migration and invasion after the overexpression of ST3GAL1 in ovarian cancer 26. However, Yoon et al. have suggested that EPHB6 induces CDH11 expression and promotes proliferation, metastasis and PTX resistance through CDH11/RhoA/FAK signaling axis 21. The explanation for the discrepancy of CDH11 function in metastasis and PTX resistance may due to cadherins function differently among distinct cell types and tissues 27. Diverse experimental conditions and inter-cohort variation may account for other reasons. And these conflicting reports promote an urgent need to explore the biological function of CDH11 in tumors.

To date, however, the expression pattern and especially the potential biological function of CDH11 in human GC with PM have not been elucidated. For the first time, the present data indicated that the downregulation of CDH11 in MKN45P cells led to the downregulation of E-cadherin, metastasis promotion and the acquisition of PTX resistance. Furthermore, the *in vivo* experiments indicated that PTX-resistant cells facilitated peritoneal metastatic nodule dissemination and resistant to PTX treatment. Moreover, inhibition of CDH11 promoted the formation of peritoneal metastatic nodules and PTX resistance. As a member of the cadherin superfamily, CDH11 is responsible for the formation of the cadherin junctions in most cell types, and the loss of cell-cell adhesion often leads to enhanced migration, invasion and metastasis 28, 29, which could in part explain why the downregulation of CDH11 promoted invasion, migration and PTX resistance in GC patients with PM. However, the exact mechanisms that by which the dysregulation of CDH11 expression mediates metastasis and PTX resistance have to be further explored.

## Conclusions

In summary, our results showed that CDH11 downregulation in PTX-resistant human ascites, tissues and cell line. Inhibition of CDH11 expression promoted the invasion, migration and PTX resistance of MKN45P cells, while overexpression of CDH11 repressed these biological functions. And CDH11 has the potential to serve as a predictable marker for the relapsed metastasis and PTX resistance in GC patients with PM.

## Supplementary Material

Supplementary figure and tables.

## Abbreviations

GC: Gastric cancer
PTX: Paclitaxel
IP: Intraperitoneal
PM: Peritoneal metastasis
TMT: Tandem Mass Tag
IHC: Immunohistochemistry
CRS: Cytoreductive surgery
HIPEC: Hyperthermic intraperitoneal chemotherapy
FBS: fetal bovine serum
