# Supplementary material for: Downregulation of CDH11 Promotes Metastasis and Resistance to Paclitaxel in Gastric Cancer Cells
Source: J Cancer. 2021 Jan 1;12(1):65–75. doi: 10.7150/jca.48193 (PMC7738820; doi:10.7150/jca.48193)
Supplement: Supplementary file 1 — Supplementary figure and tables. [file jcav12p0065s1.pdf]

## Supplementary for

# Downregulation of CDH11 Promotes Metastasis and Resistance to Paclitaxel in Gastric Cancer Cells

Zhongyin Yang [a, 1,*], Chao Yan [a, 1], Zhenjia Yu [a], Changyu He [a], Jianfang Li [a], Chen Li [a], Min Yan [a], Bingya Liu [a], Yingli Wu [b,*], Zhenggang Zhu [a,*]

# Supplementary Figure

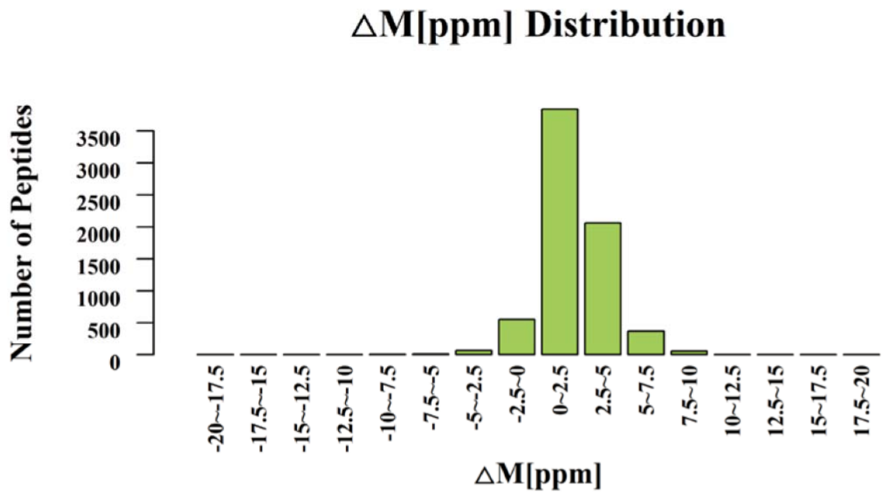


**Supplementary Figure S1.** The mass deviation of all peptides. △M: Mass deviation.

# Supplementary Tables

Table S1. Characteristics of patients

| **Symbol of patients before chemotherapy** | **Sex** | **Age** | **PCI** | **Volume of ascites (ml)** | **Cycles before relapse** | **Sustain time before relapse (month)** | **Symbol of patients after chemotherapy** |
|---|---|---|---|---|---|---|---|
| a1 | Male | 40 | 10 | 700 | 6 | 5.5 | b1 |
| a2 | Female | 31 | 36 | 200 | 8 | 6 | b2 |
| a3 | Female | 46 | 39 | 15000 | 5 | 5 | b3 |

PCI: peritoneal cancer index

Table S2. Proteins identified by TMT-based proteomics

| Accession | Gene Name | Coverage | Peptides | PSMs | Unique Peptides | AAs | MW [kDa] | calc. pI | P value |
|---|---|---|---|---|---|---|---|---|---|
| Q13421 | MSLN | 14.9 | 9 | 30 | 9 | 630 | 68.9 | 6.38 | 0.021 |
| P25940 | COL5A3 | 1.1 | 1 | 2 | 1 | 1745 | 172.0 | 6.87 | 0.018 |
| Q14767 | LTBP2 | 4.2 | 6 | 7 | 6 | 1821 | 194.9 | 5.19 | 0.042 |
| Q9UBX7 | KLK11 | 9.9 | 3 | 4 | 3 | 282 | 31.0 | 8.94 | 0.046 |
| Q9Y240 | CLEC11A | 13.9 | 4 | 4 | 4 | 323 | 35.7 | 5.16 | 0.023 |
| P55287 | CDH11 | 8.7 | 7 | 14 | 7 | 796 | 87.9 | 4.91 | 0.025 |
| P22676 | CALB2 | 4.1 | 1 | 1 | 1 | 271 | 31.5 | 5.15 | 0.037 |
| Q14314 | FGL2 | 12.5 | 4 | 8 | 4 | 439 | 50.2 | 7.39 | 0.042 |
| P07437 | TUBB | 6.1 | 2 | 2 | 2 | 444 | 49.6 | 4.89 | 0.049 |
| P25774 | CTSS | 10.3 | 3 | 3 | 3 | 331 | 37.5 | 8.34 | 0.046 |
| Q13201 | MMRN1 | 1.6 | 2 | 6 | 2 | 1228 | 138.0 | 7.93 | 0.035 |
| Q6UXS9 | CASP12 | 6.2 | 1 | 3 | 1 | 341 | 38.8 | 6.02 | 0.014 |
| Q96CX2 | KCTD12 | 3.7 | 1 | 1 | 1 | 325 | 35.7 | 5.64 | 0.022 |
| P06727 | APOA4 | 82.3 | 35 | 419 | 35 | 396 | 45.4 | 5.38 | 0.014 |
| O15204 | ADAMDEC1 | 3.6 | 1 | 1 | 1 | 470 | 52.7 | 7.34 | 0.019 |
| P68363 | TUBA1B | 20.6 | 7 | 10 | 7 | 451 | 50.1 | 5.06 | 0.002 |
| Q5HYA8 | TMEM67 | 1.4 | 1 | 2 | 1 | 995 | 111.7 | 6.77 | 0.049 |
| Q9Y5C1 | ANGPTL3 | 2.4 | 1 | 1 | 1 | 460 | 53.6 | 6.7 | 0.044 |
| Q01995 | TAGLN | 43.8 | 8 | 16 | 8 | 201 | 22.6 | 8.84 | 0.012 |
| P68871 | HBB | 83.0 | 12 | 267 | 6 | 147 | 16.0 | 7.28 | 0.373 |
| P69905 | HBA1 | 71.8 | 10 | 132 | 10 | 142 | 15.2 | 8.68 | 0.374 |
| P32119 | PRDX2 | 54.5 | 10 | 65 | 9 | 198 | 21.9 | 5.97 | 0.379 |
| P55327 | TPD52 | 6.7 | 1 | 1 | 1 | 224 | 24.3 | 4.83 | 0.343 |
| P00738 | HP | 50.5 | 21 | 136 | 21 | 406 | 45.2 | 6.58 | 0.133 |
| P02042 | HBD | 83.0 | 11 | 155 | 5 | 147 | 16.0 | 8.05 | 0.371 |
| Q5T619 | ZNF648 | 2.1 | 1 | 17 | 1 | 568 | 62.3 | 8.62 | 0.374 |
| P28330 | ACADL | 4.4 | 1 | 3 | 1 | 430 | 47.6 | 7.8 | 0.391 |
| P00915 | CA1 | 47.1 | 11 | 76 | 11 | 261 | 28.9 | 7.12 | 0.363 |
| P00918 | CA2 | 42.3 | 9 | 20 | 9 | 260 | 29.2 | 7.4 | 0.370 |
| P07910 | HNRNPC | 11.1 | 4 | 7 | 4 | 306 | 33.7 | 5.08 | 0.384 |
| P10451 | SPP1 | 23.6 | 5 | 44 | 5 | 314 | 35.4 | 4.58 | 0.399 |
| P29508 | SERPINB3 | 15.6 | 6 | 10 | 6 | 390 | 44.5 | 6.81 | 0.344 |
| P03956 | MMP1 | 4.3 | 2 | 2 | 2 | 469 | 54.0 | 6.96 | 0.435 |
| O95994 | AGR2 | 9.7 | 1 | 1 | 1 | 175 | 20.0 | 9 | 0.308 |
| Q06141 | REG3A | 31.4 | 4 | 13 | 4 | 175 | 19.4 | 7.64 | 0.444 |
| P14174 | MIF | 7.8 | 1 | 2 | 1 | 115 | 12.5 | 7.88 | 0.381 |
| P30041 | PRDX6 | 51.3 | 9 | 18 | 9 | 224 | 25.0 | 6.38 | 0.372 |
| P10599 | TXN | 40.0 | 5 | 11 | 5 | 105 | 11.7 | 4.92 | 0.363 |
| P69891 | HBG1 | 64.6 | 9 | 48 | 1 | 147 | 16.1 | 7.2 | 0.381 |

| | | | | | | | | | |
|---|---|---|---|---|---|---|---|---|---|
| P02652 | APOA2 | 68.0 | 7 | 104 | 7 | 100 | 11.2 | 6.62 | 0.416 |
| P25815 | S100P | 10.5 | 1 | 3 | 1 | 95 | 10.4 | 4.88 | 0.330 |
| P04040 | CAT | 50.7 | 22 | 62 | 22 | 527 | 59.7 | 7.39 | 0.379 |
| P08779 | KRT16 | 15.2 | 7 | 14 | 1 | 473 | 51.2 | 5.05 | 0.407 |
| P05787 | KRT8 | 22.8 | 13 | 25 | 8 | 483 | 53.7 | 5.59 | 0.388 |
| Q13404 | UBE2V1 | 6.8 | 1 | 1 | 1 | 147 | 16.5 | 7.93 | 0.432 |
| P06744 | GPI | 16.5 | 9 | 20 | 9 | 558 | 63.1 | 8.32 | 0.378 |
| Q13813 | SPTAN1 | 10.1 | 25 | 34 | 25 | 2472 | 284.4 | 5.35 | 0.369 |
| Q96C19 | EFHD2 | 4.2 | 1 | 2 | 1 | 240 | 26.7 | 5.2 | 0.443 |
| P15531 | NME1 | 23.0 | 3 | 3 | 1 | 152 | 17.1 | 6.19 | 0.360 |
| Q01518 | CAP1 | 7.8 | 3 | 6 | 3 | 475 | 51.9 | 8.06 | 0.385 |
| Q9H0N5 | PCBD2 | 5.4 | 1 | 1 | 1 | 130 | 14.4 | 9.11 | 0.410 |
| P09466 | PAEP | 3.9 | 1 | 1 | 1 | 180 | 20.6 | 5.57 | 0.278 |
| Q06830 | PRDX1 | 42.2 | 9 | 30 | 8 | 199 | 22.1 | 8.13 | 0.403 |
| Q08174 | PCDH1 | 1.4 | 1 | 1 | 1 | 1060 | 114.7 | 5.03 | 0.249 |
| O14818 | PSMA7 | 12.9 | 3 | 3 | 3 | 248 | 27.9 | 8.46 | 0.382 |
| P26447 | S100A4 | 15.8 | 2 | 5 | 2 | 101 | 11.7 | 6.11 | 0.450 |
| Q9BZH6 | WDR11 | 0.7 | 1 | 1 | 1 | 1224 | 136.6 | 6.92 | 0.434 |
| P31949 | S100A11 | 42.9 | 4 | 7 | 4 | 105 | 11.7 | 7.12 | 0.426 |
| P69892 | HBG2 | 64.6 | 9 | 45 | 1 | 147 | 16.1 | 7.2 | 0.415 |
| P13667 | PDIA4 | 27.0 | 15 | 25 | 15 | 645 | 72.9 | 5.07 | 0.368 |
| P12107 | COL11A1 | 1.2 | 1 | 1 | 1 | 1806 | 181.0 | 5.17 | 0.153 |
| P07148 | FABP1 | 50.4 | 5 | 10 | 5 | 127 | 14.2 | 7.18 | 0.460 |
| Q6UX06 | OLFM4 | 8.4 | 4 | 5 | 4 | 510 | 57.2 | 5.69 | 0.331 |
| Q16851 | UGP2 | 24.0 | 9 | 14 | 9 | 508 | 56.9 | 8.15 | 0.382 |
| P04632 | CAPNS1 | 2.6 | 1 | 1 | 1 | 268 | 28.3 | 5.2 | 0.370 |
| P51858 | HDGF | 11.3 | 2 | 3 | 2 | 240 | 26.8 | 4.73 | 0.356 |
| P02549 | SPTA1 | 1.9 | 5 | 5 | 5 | 2419 | 279.8 | 5.05 | 0.370 |
| P02730 | SLC4A1 | 4.7 | 3 | 4 | 3 | 911 | 101.7 | 5.19 | 0.400 |
| P15529 | CD46 | 2.3 | 1 | 1 | 1 | 392 | 43.7 | 6.74 | 0.356 |
| P02792 | FTL | 41.7 | 7 | 36 | 7 | 175 | 20.0 | 5.78 | 0.513 |
| P27797 | CALR | 24.0 | 8 | 20 | 8 | 417 | 48.1 | 4.44 | 0.394 |
| P40925 | MDH1 | 29.3 | 9 | 16 | 9 | 334 | 36.4 | 7.36 | 0.391 |
| Q86VD1 | MORC1 | 0.7 | 1 | 3 | 1 | 984 | 112.8 | 7.85 | 0.479 |
| Q8TAQ9 | SUN3 | 2.0 | 1 | 3 | 1 | 357 | 40.5 | 7.83 | 0.344 |
| P06703 | S100A6 | 24.4 | 3 | 5 | 3 | 90 | 10.2 | 5.48 | 0.418 |
| Q01082 | SPTBN1 | 1.7 | 4 | 4 | 4 | 2364 | 274.4 | 5.57 | 0.405 |
| Q15149 | PLEC | 0.2 | 1 | 1 | 1 | 4684 | 531.5 | 5.96 | 0.352 |
| P30048 | PRDX3 | 8.6 | 2 | 4 | 2 | 256 | 27.7 | 7.78 | 0.356 |
| P00492 | HPRT1 | 20.2 | 4 | 6 | 4 | 218 | 24.6 | 6.68 | 0.361 |
| P07237 | P4HB | 36.2 | 17 | 26 | 17 | 508 | 57.1 | 4.87 | 0.374 |
| P30046 | DDT | 28.8 | 4 | 10 | 4 | 118 | 12.7 | 7.3 | 0.428 |
| P14314 | PRKCSH | 10.2 | 5 | 8 | 5 | 528 | 59.4 | 4.41 | 0.440 |

| | | | | | | | | | |
|---|---|---|---|---|---|---|---|---|---|
| P02545 | LMNA | 26.1 | 17 | 26 | 17 | 664 | 74.1 | 7.02 | 0.435 |
| P00441 | SOD1 | 20.1 | 3 | 9 | 3 | 154 | 15.9 | 6.13 | 0.429 |
| P06733 | ENO1 | 45.9 | 15 | 33 | 15 | 434 | 47.1 | 7.39 | 0.370 |
| Q5SRE5 | NUP188 | 0.4 | 1 | 2 | 1 | 1749 | 195.9 | 6.73 | 0.423 |
| P00491 | PNP | 23.2 | 5 | 8 | 5 | 289 | 32.1 | 6.95 | 0.409 |
| Q14508 | WFDC2 | 32.3 | 3 | 5 | 3 | 124 | 13.0 | 4.84 | 0.393 |
| P04406 | GAPDH | 43.6 | 11 | 24 | 11 | 335 | 36.0 | 8.46 | 0.411 |
| Q4ZHG4 | FNDC1 | 1.6 | 2 | 3 | 2 | 1894 | 205.4 | 9.32 | 0.161 |
| P62979 | RPS27A | 16.0 | 2 | 4 | 2 | 156 | 18.0 | 9.64 | 0.414 |
| P0DP25 | CALM3 | 42.3 | 5 | 14 | 5 | 149 | 16.8 | 4.22 | 0.488 |
| Q15257 | PTPA | 2.2 | 1 | 1 | 1 | 358 | 40.6 | 5.94 | 0.350 |
| Q99497 | PARK7 | 41.8 | 6 | 8 | 6 | 189 | 19.9 | 6.79 | 0.417 |
| P17174 | GOT1 | 24.0 | 9 | 16 | 9 | 413 | 46.2 | 7.01 | 0.438 |
| P28070 | PSMB4 | 7.6 | 1 | 2 | 1 | 264 | 29.2 | 5.97 | 0.310 |
| P28066 | PSMA5 | 25.3 | 5 | 9 | 5 | 241 | 26.4 | 4.79 | 0.427 |
| Q06481 | APLP2 | 5.5 | 4 | 5 | 3 | 763 | 86.9 | 4.79 | 0.475 |
| P40306 | PSMB10 | 6.2 | 2 | 2 | 2 | 273 | 28.9 | 7.81 | 0.243 |
| Q68BL8 | OLFML2B | 1.3 | 1 | 1 | 1 | 750 | 83.9 | 5.2 | 0.189 |
| P02794 | FTH1 | 39.9 | 5 | 11 | 5 | 183 | 21.2 | 5.55 | 0.445 |
| P23528 | CFL1 | 37.3 | 5 | 5 | 5 | 166 | 18.5 | 8.09 | 0.365 |
| Q96C23 | GALM | 5.0 | 1 | 1 | 1 | 342 | 37.7 | 6.65 | 0.459 |
| P00558 | PGK1 | 28.1 | 10 | 14 | 10 | 417 | 44.6 | 8.1 | 0.419 |
| Q03403 | TFF2 | 21.7 | 2 | 3 | 2 | 129 | 14.3 | 5.81 | 0.532 |
| P63208 | SKP1 | 4.9 | 1 | 2 | 1 | 163 | 18.6 | 4.54 | 0.433 |
| Q9BS40 | LXN | 4.1 | 1 | 1 | 1 | 222 | 25.7 | 5.78 | 0.444 |
| O43278 | SPINT1 | 11.9 | 6 | 8 | 6 | 529 | 58.4 | 6.29 | 0.327 |
| Q9UGM3 | DMBT1 | 22.0 | 5 | 13 | 5 | 2413 | 260.6 | 5.44 | 0.426 |
| Q99436 | PSMB7 | 10.8 | 3 | 3 | 3 | 277 | 29.9 | 7.68 | 0.456 |
| Q13228 | SELENBP1 | 18.2 | 8 | 13 | 8 | 472 | 52.4 | 6.37 | 0.424 |
| Q9P2E9 | RRBP1 | 0.9 | 1 | 1 | 1 | 1410 | 152.4 | 8.6 | 0.431 |
| P12277 | CKB | 4.5 | 1 | 2 | 1 | 381 | 42.6 | 5.59 | 0.598 |
| Q99715 | COL12A1 | 11.5 | 27 | 38 | 27 | 3063 | 332.9 | 5.53 | 0.150 |
| P25788 | PSMA3 | 6.3 | 2 | 2 | 2 | 255 | 28.4 | 5.33 | 0.432 |
| P01024 | C3 | 60.7 | 87 | 491 | 87 | 1663 | 187.0 | 6.4 | 0.344 |
| P49720 | PSMB3 | 19.5 | 3 | 3 | 3 | 205 | 22.9 | 6.55 | 0.355 |
| P07738 | BPGM | 18.5 | 4 | 7 | 4 | 259 | 30.0 | 6.54 | 0.477 |
| P50395 | GDI2 | 31.0 | 11 | 19 | 10 | 445 | 50.6 | 6.47 | 0.438 |
| P22223 | CDH3 | 3.0 | 2 | 3 | 1 | 829 | 91.4 | 4.75 | 0.280 |
| Q15084 | PDIA6 | 15.9 | 5 | 8 | 5 | 440 | 48.1 | 5.08 | 0.411 |
| Q8N130 | SLC34A3 | 1.3 | 1 | 3 | 1 | 599 | 63.5 | 8.27 | 0.273 |
| P30740 | SERPINB1 | 23.2 | 8 | 13 | 8 | 379 | 42.7 | 6.28 | 0.415 |
| Q15063 | POSTN | 51.3 | 35 | 165 | 35 | 836 | 93.3 | 7.53 | 0.414 |
| Q15582 | TGFBI | 44.2 | 24 | 86 | 24 | 683 | 74.6 | 7.71 | 0.260 |

| | | | | | | | | | |
|---|---|---|---|---|---|---|---|---|---|
| O00592 | PODXL | 3.9 | 2 | 2 | 2 | 558 | 58.6 | 5.49 | 0.226 |
| P02538 | KRT6A | 17.6 | 10 | 18 | 1 | 564 | 60.0 | 8 | 0.498 |
| Q5JXB2 | UBE2NL | 6.5 | 1 | 1 | 1 | 153 | 17.4 | 5.92 | 0.409 |
| Q9NUQ9 | FAM49B | 3.1 | 1 | 1 | 1 | 324 | 36.7 | 6.06 | 0.120 |
| P60174 | TPI1 | 56.6 | 12 | 27 | 12 | 286 | 30.8 | 5.92 | 0.425 |
| P05783 | KRT18 | 7.0 | 3 | 3 | 2 | 430 | 48.0 | 5.45 | 0.468 |
| P35579 | MYH9 | 2.9 | 5 | 8 | 5 | 1960 | 226.4 | 5.6 | 0.496 |
| P05423 | POLR3D | 1.8 | 1 | 2 | 1 | 398 | 44.4 | 6.98 | 0.105 |
| P13611 | VCAN | 4.2 | 13 | 26 | 13 | 3396 | 372.6 | 4.51 | 0.216 |
| P60900 | PSMA6 | 17.5 | 4 | 8 | 4 | 246 | 27.4 | 6.76 | 0.387 |
| P05997 | COL5A2 | 6.3 | 7 | 11 | 7 | 1499 | 144.8 | 6.46 | 0.126 |
| P11142 | HSPA8 | 20.1 | 12 | 20 | 7 | 646 | 70.9 | 5.52 | 0.460 |
| P15311 | EZR | 35.3 | 20 | 34 | 15 | 586 | 69.4 | 6.27 | 0.429 |
| P25786 | PSMA1 | 19.8 | 5 | 7 | 5 | 263 | 29.5 | 6.61 | 0.449 |
| P29966 | MARCKS | 3.3 | 1 | 2 | 1 | 332 | 31.5 | 4.45 | 0.550 |
| P62258 | YWHAE | 42.4 | 9 | 18 | 6 | 255 | 29.2 | 4.74 | 0.478 |
| P04424 | ASL | 8.6 | 3 | 5 | 3 | 464 | 51.6 | 6.48 | 0.347 |
| P04080 | CSTB | 12.2 | 1 | 2 | 1 | 98 | 11.1 | 7.56 | 0.480 |
| Q9NR99 | MXRA5 | 7.0 | 16 | 25 | 16 | 2828 | 312.0 | 8.32 | 0.247 |
| P02787 | TF | 37.7 | 24 | 52 | 24 | 698 | 77.0 | 7.12 | 0.274 |
| P19652 | ORM2 | 8.5 | 1 | 1 | 1 | 201 | 23.6 | 5.11 | 0.414 |
| P09211 | GSTP1 | 34.8 | 5 | 11 | 5 | 210 | 23.3 | 5.64 | 0.397 |
| P28065 | PSMB9 | 8.7 | 2 | 2 | 2 | 219 | 23.3 | 5.03 | 0.385 |
| P22392 | NME2 | 23.0 | 3 | 3 | 1 | 152 | 17.3 | 8.41 | 0.499 |
| P00325 | ADH1B | 2.1 | 1 | 1 | 1 | 375 | 39.8 | 8.29 | 0.073 |
| Q08380 | LGALS3BP | 32.8 | 15 | 65 | 15 | 585 | 65.3 | 5.27 | 0.301 |
| O43707 | ACTN4 | 25.9 | 19 | 26 | 11 | 911 | 104.8 | 5.44 | 0.416 |
| P00338 | LDHA | 22.3 | 7 | 22 | 6 | 332 | 36.7 | 8.27 | 0.445 |
| P0DOX5 | | 27.6 | 10 | 51 | 5 | 449 | 49.3 | 8.72 | 0.452 |
| P20700 | LMNB1 | 3.8 | 2 | 2 | 2 | 586 | 66.4 | 5.16 | 0.345 |
| P20142 | PGC | 2.1 | 1 | 1 | 1 | 388 | 42.4 | 4.46 | 0.497 |
| Q9UL46 | PSME2 | 3.8 | 1 | 1 | 1 | 239 | 27.4 | 5.73 | 0.438 |
| Q06323 | PSME1 | 8.0 | 2 | 2 | 2 | 249 | 28.7 | 6.02 | 0.543 |
| P20618 | PSMB1 | 17.8 | 3 | 4 | 3 | 241 | 26.5 | 8.13 | 0.298 |
| P47972 | NPTX2 | 2.8 | 1 | 1 | 1 | 431 | 47.0 | 5.63 | 0.406 |
| P07384 | CAPN1 | 1.1 | 1 | 1 | 1 | 714 | 81.8 | 5.67 | 0.336 |
| P52209 | PGD | 2.3 | 1 | 1 | 1 | 483 | 53.1 | 7.23 | 0.452 |
| P17936 | IGFBP3 | 33.3 | 10 | 33 | 10 | 291 | 31.7 | 8.69 | 0.205 |
| P20908 | COL5A1 | 11.6 | 15 | 47 | 15 | 1838 | 183.4 | 5.06 | 0.189 |
| P31946 | YWHAB | 29.3 | 7 | 12 | 2 | 246 | 28.1 | 4.83 | 0.503 |
| O14950 | MYL12B | 16.3 | 2 | 2 | 2 | 172 | 19.8 | 4.84 | 0.496 |
| P25789 | PSMA4 | 30.7 | 6 | 11 | 6 | 261 | 29.5 | 7.72 | 0.468 |
| P40121 | CAPG | 14.1 | 4 | 5 | 4 | 348 | 38.5 | 6.19 | 0.493 |

| | | | | | | | | | |
|---|---|---|---|---|---|---|---|---|---|
| P46940 | IQGAP1 | 2.2 | 2 | 2 | 2 | 1657 | 189.1 | 6.48 | 0.396 |
| P18827 | SDC1 | 5.5 | 1 | 4 | 1 | 310 | 32.4 | 4.63 | 0.479 |
| P25787 | PSMA2 | 20.9 | 3 | 4 | 3 | 234 | 25.9 | 7.43 | 0.096 |
| P02768 | ALB | 71.1 | 44 | 209 | 44 | 609 | 69.3 | 6.28 | 0.140 |
| P02775 | PPBP | 38.3 | 4 | 8 | 4 | 128 | 13.9 | 8.79 | 0.527 |
| Q8WUT4 | LRRN4 | 2.7 | 2 | 5 | 2 | 740 | 78.8 | 7.2 | 0.159 |
| P14649 | MYL6B | 5.3 | 1 | 1 | 1 | 208 | 22.8 | 5.73 | 0.318 |
| P20810 | CAST | 6.1 | 3 | 3 | 3 | 708 | 76.5 | 5.07 | 0.600 |
| Q9NZP8 | C1RL | 31.6 | 13 | 47 | 11 | 487 | 53.5 | 7.2 | 0.285 |
| P00995 | SPINK1 | 10.1 | 1 | 1 | 1 | 79 | 8.5 | 7.61 | 0.197 |
| P00352 | ALDH1A1 | 7.0 | 3 | 3 | 3 | 501 | 54.8 | 6.73 | 0.507 |
| P0DMV9 | HSPA1B | 15.1 | 9 | 14 | 5 | 641 | 70.0 | 5.66 | 0.381 |
| P46926 | GNPDA1 | 7.6 | 2 | 2 | 2 | 289 | 32.6 | 6.92 | 0.470 |
| P30101 | PDIA3 | 17.2 | 8 | 12 | 8 | 505 | 56.7 | 6.35 | 0.353 |
| Q9H1E3 | NUCKS1 | 3.7 | 1 | 1 | 1 | 243 | 27.3 | 5.08 | 0.434 |
| P99999 | CYCS | 18.1 | 2 | 2 | 2 | 105 | 11.7 | 9.57 | 0.435 |
| P07900 | HSP90AA1 | 18.4 | 11 | 17 | 7 | 732 | 84.6 | 5.02 | 0.432 |
| P08397 | HMBS | 4.7 | 1 | 1 | 1 | 361 | 39.3 | 7.18 | 0.617 |
| P13716 | ALAD | 27.3 | 7 | 10 | 7 | 330 | 36.3 | 6.79 | 0.342 |
| P61077 | UBE2D3 | 7.5 | 1 | 1 | 1 | 147 | 16.7 | 7.8 | 0.430 |
| P31431 | SDC4 | 12.1 | 2 | 4 | 2 | 198 | 21.6 | 4.5 | 0.291 |
| P67936 | TPM4 | 35.5 | 10 | 18 | 5 | 248 | 28.5 | 4.69 | 0.550 |
| Q9HC38 | GLOD4 | 9.9 | 3 | 3 | 3 | 313 | 34.8 | 5.6 | 0.542 |
| P01009 | SERPINA1 | 33.7 | 13 | 26 | 13 | 418 | 46.7 | 5.59 | 0.474 |
| P08833 | IGFBP1 | 11.6 | 2 | 2 | 2 | 259 | 27.9 | 5.19 | 0.454 |
| P00390 | GSR | 10.3 | 5 | 8 | 5 | 522 | 56.2 | 8.5 | 0.481 |
| Q16568 | CARTPT | 12.9 | 1 | 1 | 1 | 116 | 12.8 | 8.25 | 0.198 |
| P01718 | IGLV3-27 | 8.8 | 1 | 1 | 1 | 113 | 12.2 | 5.01 | 0.470 |
| Q12907 | LMAN2 | 27.5 | 7 | 14 | 7 | 356 | 40.2 | 6.95 | 0.504 |
| P39059 | COL15A1 | 2.1 | 3 | 3 | 3 | 1388 | 141.6 | 5 | 0.330 |
| O75347 | TBCA | 6.5 | 1 | 1 | 1 | 108 | 12.8 | 5.29 | 0.591 |
| P35237 | SERPINB6 | 9.6 | 3 | 5 | 3 | 376 | 42.6 | 5.27 | 0.599 |
| A1L4H1 | SSC5D | 17.7 | 16 | 23 | 16 | 1573 | 165.6 | 6.13 | 0.467 |
| P07339 | CTSD | 37.9 | 11 | 36 | 11 | 412 | 44.5 | 6.54 | 0.565 |
| P37837 | TALDO1 | 25.5 | 8 | 12 | 8 | 337 | 37.5 | 6.81 | 0.517 |
| Q93045 | STMN2 | 5.0 | 1 | 1 | 1 | 179 | 20.8 | 8.32 | 0.364 |
| Q9NY33 | DPP3 | 2.7 | 1 | 1 | 1 | 737 | 82.5 | 5.1 | 0.342 |
| Q02985 | CFHR3 | 6.1 | 2 | 28 | 1 | 330 | 37.3 | 7.55 | 0.501 |
| Q04760 | GLO1 | 4.9 | 1 | 1 | 1 | 184 | 20.8 | 5.31 | 0.601 |
| O00468 | AGRN | 8.2 | 13 | 17 | 13 | 2067 | 217.1 | 6.39 | 0.340 |
| P08758 | ANXA5 | 10.6 | 3 | 4 | 3 | 320 | 35.9 | 5.05 | 0.603 |
| P22626 | HNRNPA2B1 | 6.5 | 2 | 2 | 2 | 353 | 37.4 | 8.95 | 0.402 |
| P35858 | IGFALS | 40.0 | 20 | 126 | 20 | 605 | 66.0 | 6.79 | 0.337 |

| | | | | | | | | | |
|---|---|---|---|---|---|---|---|---|---|
| P36222 | CHI3L1 | 40.7 | 12 | 29 | 12 | 383 | 42.6 | 8.46 | 0.189 |
| Q8NBJ4 | GOLM1 | 33.2 | 12 | 27 | 12 | 401 | 45.3 | 4.97 | 0.191 |
| Q9Y5Y6 | ST14 | 0.9 | 1 | 1 | 1 | 855 | 94.7 | 6.55 | 0.446 |
| P12814 | ACTN1 | 13.6 | 10 | 17 | 2 | 892 | 103.0 | 5.41 | 0.721 |
| O00584 | RNASET2 | 20.7 | 4 | 7 | 4 | 256 | 29.5 | 7.08 | 0.407 |
| Q562R1 | ACTBL2 | 14.9 | 4 | 28 | 1 | 376 | 42.0 | 5.59 | 0.255 |
| O15389 | SIGLEC5 | 1.3 | 1 | 1 | 1 | 551 | 60.7 | 7.27 | 0.800 |
| P04075 | ALDOA | 35.7 | 12 | 19 | 10 | 364 | 39.4 | 8.09 | 0.467 |
| O75326 | SEMA7A | 2.3 | 1 | 1 | 1 | 666 | 74.8 | 7.64 | 0.438 |
| P09486 | SPARC | 39.9 | 11 | 35 | 11 | 303 | 34.6 | 4.84 | 0.460 |
| Q92954 | PRG4 | 12.4 | 16 | 46 | 16 | 1404 | 151.0 | 9.51 | 0.309 |
| P06702 | S100A9 | 44.7 | 5 | 9 | 5 | 114 | 13.2 | 6.13 | 0.571 |
| P43652 | AFM | 61.8 | 35 | 376 | 35 | 599 | 69.0 | 5.9 | 0.348 |
| P04216 | THY1 | 18.0 | 2 | 4 | 2 | 161 | 17.9 | 8.73 | 0.555 |
| P30044 | PRDX5 | 5.1 | 1 | 1 | 1 | 214 | 22.1 | 8.7 | 0.381 |
| P98172 | EFNB1 | 5.8 | 2 | 3 | 2 | 346 | 38.0 | 8.94 | 0.335 |
| P63104 | YWHAZ | 53.1 | 11 | 20 | 7 | 245 | 27.7 | 4.79 | 0.525 |
| P07195 | LDHB | 37.7 | 12 | 40 | 11 | 334 | 36.6 | 6.05 | 0.585 |
| P11597 | CETP | 3.0 | 1 | 1 | 1 | 493 | 54.7 | 6.09 | 0.373 |
| Q9BRA2 | TXNDC17 | 7.3 | 1 | 1 | 1 | 123 | 13.9 | 5.52 | 0.474 |
| Q14956 | GPNMB | 4.7 | 2 | 7 | 2 | 572 | 63.9 | 6.64 | 0.397 |
| P49746 | THBS3 | 7.9 | 6 | 9 | 5 | 956 | 104.1 | 4.65 | 0.475 |
| P02461 | COL3A1 | 7.5 | 9 | 34 | 9 | 1466 | 138.5 | 6.61 | 0.590 |
| P02452 | COL1A1 | 15.8 | 18 | 55 | 18 | 1464 | 138.9 | 5.8 | 0.510 |
| P49257 | LMAN1 | 2.9 | 1 | 1 | 1 | 510 | 57.5 | 6.77 | 0.346 |
| P08697 | SERPINF2 | 47.3 | 22 | 182 | 22 | 491 | 54.5 | 6.29 | 0.635 |
| Q01469 | FABP5 | 10.4 | 1 | 1 | 1 | 135 | 15.2 | 7.01 | 0.254 |
| P13727 | PRG2 | 3.2 | 1 | 1 | 1 | 222 | 25.2 | 6.76 | 0.493 |
| P42126 | ECI1 | 5.0 | 1 | 1 | 1 | 302 | 32.8 | 8.54 | 0.458 |
| P61981 | YWHAG | 34.4 | 8 | 13 | 4 | 247 | 28.3 | 4.89 | 0.572 |
| Q02818 | NUCB1 | 30.4 | 11 | 22 | 11 | 461 | 53.8 | 5.25 | 0.219 |
| P22891 | PROZ | 18.8 | 8 | 22 | 8 | 400 | 44.7 | 5.97 | 0.545 |
| O00299 | CLIC1 | 16.2 | 3 | 3 | 3 | 241 | 26.9 | 5.17 | 0.183 |
| P02679 | FGG | 71.7 | 28 | 259 | 28 | 453 | 51.5 | 5.62 | 0.708 |
| O95336 | PGLS | 3.1 | 1 | 1 | 1 | 258 | 27.5 | 6.05 | 0.195 |
| Q92692 | NECTIN2 | 3.5 | 2 | 2 | 2 | 538 | 57.7 | 4.82 | 0.309 |
| P26038 | MSN | 38.1 | 22 | 39 | 17 | 577 | 67.8 | 6.4 | 0.667 |
| O75339 | CILP | 9.7 | 10 | 12 | 10 | 1184 | 132.5 | 8.41 | 0.351 |
| Q9GZT8 | NIF3L1 | 6.1 | 2 | 2 | 2 | 377 | 41.9 | 6.65 | 0.573 |
| P12109 | COL6A1 | 19.3 | 17 | 66 | 17 | 1028 | 108.5 | 5.43 | 0.246 |
| Q13867 | BLMH | 9.5 | 4 | 5 | 4 | 455 | 52.5 | 6.27 | 0.565 |
| P02763 | ORM1 | 7.5 | 1 | 1 | 1 | 201 | 23.5 | 5.02 | 0.382 |
| O94985 | CLSTN1 | 16.0 | 15 | 29 | 15 | 981 | 109.7 | 4.91 | 0.307 |

| | | | | | | | | | |
|---|---|---|---|---|---|---|---|---|---|
| Q13443 | ADAM9 | 1.2 | 1 | 1 | 1 | 819 | 90.5 | 7.52 | 0.652 |
| P06276 | BCHE | 35.0 | 17 | 68 | 17 | 602 | 68.4 | 7.42 | 0.293 |
| P30086 | PEBP1 | 30.5 | 4 | 8 | 4 | 187 | 21.0 | 7.53 | 0.567 |
| Q9NP78 | ABCB9 | 0.9 | 1 | 6 | 1 | 766 | 84.4 | 7.93 | 0.665 |
| P29692 | EEF1D | 4.3 | 1 | 1 | 1 | 281 | 31.1 | 5.01 | 0.586 |
| P19338 | NCL | 1.1 | 1 | 1 | 1 | 710 | 76.6 | 4.7 | 0.624 |
| Q8IXL6 | FAM20C | 8.2 | 3 | 3 | 3 | 584 | 66.2 | 7.74 | 0.351 |
| Q96GP6 | SCARF2 | 1.4 | 1 | 1 | 1 | 870 | 92.4 | 8.44 | 0.534 |
| P11021 | HSPA5 | 35.9 | 19 | 34 | 16 | 654 | 72.3 | 5.16 | 0.210 |
| P02743 | APCS | 33.2 | 8 | 47 | 8 | 223 | 25.4 | 6.54 | 0.440 |
| P05154 | SERPINA5 | 35.0 | 14 | 68 | 14 | 406 | 45.6 | 9.26 | 0.307 |
| P28799 | GRN | 10.8 | 5 | 6 | 5 | 593 | 63.5 | 6.83 | 0.401 |
| P01764 | IGHV3-23 | 9.4 | 1 | 2 | 1 | 117 | 12.6 | 8.28 | 0.558 |
| Q9BXR6 | CFHR5 | 10.0 | 4 | 12 | 3 | 569 | 64.4 | 7.06 | 0.602 |
| P16152 | CBR1 | 10.1 | 2 | 2 | 2 | 277 | 30.4 | 8.32 | 0.534 |
| Q15262 | PTPRK | 0.5 | 1 | 1 | 1 | 1439 | 162.0 | 5.9 | 0.553 |
| P01019 | AGT | 31.3 | 12 | 165 | 12 | 485 | 53.1 | 6.32 | 0.390 |
| P01860 | IGHG3 | 48.0 | 13 | 108 | 6 | 377 | 41.3 | 7.9 | 0.790 |
| Q92626 | PXDN | 1.2 | 1 | 1 | 1 | 1479 | 165.2 | 7.17 | 0.493 |
| P02675 | FGB | 70.7 | 29 | 420 | 29 | 491 | 55.9 | 8.27 | 0.722 |
| Q96A08 | HIST1H2BA | 7.1 | 1 | 1 | 1 | 127 | 14.2 | 10.3 | 0.619 |
| Q9UI42 | CPA4 | 10.7 | 3 | 3 | 3 | 421 | 47.3 | 6.7 | 0.454 |
| P06454 | PTMA | 12.6 | 1 | 1 | 1 | 111 | 12.2 | 3.78 | 0.326 |
| P16930 | FAH | 12.6 | 5 | 11 | 5 | 419 | 46.3 | 6.95 | 0.549 |
| P04156 | PRNP | 3.6 | 1 | 2 | 1 | 253 | 27.6 | 9 | 0.771 |
| Q96PD2 | DCBLD2 | 5.2 | 3 | 3 | 3 | 775 | 85.0 | 7.17 | 0.211 |
| P08123 | COL1A2 | 11.1 | 13 | 32 | 13 | 1366 | 129.2 | 8.95 | 0.596 |
| P07602 | PSAP | 24.8 | 12 | 25 | 12 | 524 | 58.1 | 5.17 | 0.751 |
| P07996 | THBS1 | 18.0 | 18 | 34 | 18 | 1170 | 129.3 | 4.94 | 0.496 |
| Q96KN2 | CNDP1 | 38.1 | 16 | 70 | 16 | 507 | 56.7 | 5.3 | 0.673 |
| P22792 | CPN2 | 30.3 | 14 | 111 | 14 | 545 | 60.5 | 5.99 | 0.208 |
| P24593 | IGFBP5 | 19.9 | 3 | 8 | 3 | 272 | 30.6 | 8.21 | 0.341 |
| Q08345 | DDR1 | 1.0 | 1 | 1 | 1 | 913 | 101.1 | 6.83 | 0.648 |
| P27169 | PON1 | 42.3 | 11 | 48 | 11 | 355 | 39.7 | 5.22 | 0.663 |
| P04179 | SOD2 | 22.5 | 5 | 8 | 5 | 222 | 24.7 | 8.25 | 0.741 |
| P80188 | LCN2 | 35.9 | 5 | 19 | 5 | 198 | 22.6 | 8.91 | 0.749 |
| P08727 | KRT19 | 12.8 | 5 | 10 | 2 | 400 | 44.1 | 5.14 | 0.590 |
| P11717 | IGF2R | 3.3 | 7 | 8 | 7 | 2491 | 274.2 | 5.94 | 0.054 |
| P63261 | ACTG1 | 61.1 | 17 | 103 | 1 | 375 | 41.8 | 5.48 | 0.532 |
| A6NMY6 | ANXA2P2 | 10.0 | 3 | 3 | 3 | 339 | 38.6 | 6.95 | 0.748 |
| P02790 | HPX | 58.9 | 25 | 1180 | 25 | 462 | 51.6 | 7.02 | 0.677 |
| O95479 | H6PD | 7.3 | 6 | 7 | 6 | 791 | 88.8 | 7.3 | 0.162 |
| P02751 | FN1 | 50.5 | 86 | 809 | 86 | 2386 | 262.5 | 5.71 | 0.433 |

| | | | | | | | | | |
|---|---|---|---|---|---|---|---|---|---|
| P05067 | APP | 1.9 | 2 | 2 | 1 | 770 | 86.9 | 4.82 | 0.791 |
| Q14766 | LTBP1 | 2.7 | 4 | 5 | 4 | 1721 | 186.7 | 5.96 | 0.280 |
| P13473 | LAMP2 | 4.6 | 2 | 5 | 2 | 410 | 44.9 | 5.63 | 0.272 |
| Q9NX02 | NLRP2 | 1.3 | 1 | 1 | 1 | 1062 | 120.4 | 6.07 | 0.660 |
| Q15485 | FCN2 | 2.2 | 1 | 1 | 1 | 313 | 34.0 | 6.77 | 0.772 |
| Q14126 | DSG2 | 15.3 | 14 | 37 | 14 | 1118 | 122.2 | 5.24 | 0.101 |
| P18669 | PGAM1 | 22.8 | 5 | 12 | 5 | 254 | 28.8 | 7.18 | 0.723 |
| P01859 | IGHG2 | 25.8 | 8 | 44 | 3 | 326 | 35.9 | 7.59 | 0.655 |
| P49788 | RARRES1 | 3.1 | 1 | 2 | 1 | 294 | 33.3 | 8.51 | 0.530 |
| P05546 | SERPIND1 | 48.1 | 22 | 207 | 22 | 499 | 57.0 | 6.9 | 0.410 |
| P08670 | VIM | 33.9 | 16 | 33 | 13 | 466 | 53.6 | 5.12 | 0.824 |
| P20061 | TCN1 | 11.1 | 3 | 4 | 3 | 433 | 48.2 | 5.03 | 0.811 |
| P52565 | ARHGDIA | 10.8 | 2 | 2 | 2 | 204 | 23.2 | 5.11 | 0.595 |
| P40199 | CEACAM6 | 9.9 | 2 | 3 | 2 | 344 | 37.2 | 5.82 | 0.662 |
| P35052 | GPC1 | 2.3 | 1 | 1 | 1 | 558 | 61.6 | 7.3 | 0.350 |
| P29622 | SERPINA4 | 54.6 | 21 | 189 | 21 | 427 | 48.5 | 7.75 | 0.470 |
| P98160 | HSPG2 | 21.7 | 70 | 149 | 70 | 4391 | 468.5 | 6.51 | 0.319 |
| P03952 | KLKB1 | 54.4 | 33 | 197 | 33 | 638 | 71.3 | 8.22 | 0.445 |
| O75874 | IDH1 | 21.0 | 9 | 12 | 9 | 414 | 46.6 | 7.01 | 0.745 |
| Q8NBS9 | TXNDC5 | 16.0 | 6 | 11 | 6 | 432 | 47.6 | 5.97 | 0.707 |
| Q15113 | PCOLCE | 13.4 | 4 | 7 | 4 | 449 | 47.9 | 7.43 | 0.540 |
| P07858 | CTSB | 28.3 | 8 | 23 | 8 | 339 | 37.8 | 6.3 | 0.733 |
| Q12866 | MERTK | 1.7 | 2 | 2 | 2 | 999 | 110.2 | 5.8 | 0.636 |
| P55285 | CDH6 | 3.4 | 2 | 5 | 2 | 790 | 88.3 | 4.93 | 0.175 |
| P20062 | TCN2 | 7.0 | 3 | 3 | 3 | 427 | 47.5 | 7.01 | 0.746 |
| P62805 | HIST1H4A | 19.4 | 2 | 2 | 2 | 103 | 11.4 | 11.4 | 0.570 |
| Q86WI1 | PKHD1L1 | 1.6 | 4 | 5 | 4 | 4243 | 465.4 | 6.11 | 0.521 |
| Q9H4A9 | DPEP2 | 12.6 | 5 | 7 | 5 | 486 | 53.3 | 6.54 | 0.450 |
| P62942 | FKBP1A | 12.0 | 1 | 2 | 1 | 108 | 11.9 | 8.16 | 0.668 |
| Q9H299 | SH3BGRL3 | 28.0 | 2 | 2 | 2 | 93 | 10.4 | 4.93 | 0.759 |
| P00734 | F2 | 59.0 | 35 | 579 | 35 | 622 | 70.0 | 5.9 | 0.644 |
| P02533 | KRT14 | 17.6 | 8 | 15 | 2 | 472 | 51.5 | 5.16 | 0.763 |
| P12830 | CDH1 | 14.1 | 10 | 31 | 9 | 882 | 97.4 | 4.73 | 0.598 |
| P14618 | PKM | 32.6 | 14 | 16 | 14 | 531 | 57.9 | 7.84 | 0.723 |
| P01705 | IGLV2-23 | 7.1 | 1 | 1 | 1 | 113 | 11.9 | 5.91 | 0.657 |
| P02749 | APOH | 58.8 | 16 | 312 | 16 | 345 | 38.3 | 7.97 | 0.625 |
| Q9UK55 | SERPINA10 | 35.6 | 13 | 54 | 13 | 444 | 50.7 | 8.27 | 0.532 |
| Q8N2S1 | LTBP4 | 2.5 | 3 | 4 | 3 | 1624 | 173.3 | 5.43 | 0.227 |
| Q9HCB6 | SPON1 | 11.9 | 8 | 10 | 8 | 807 | 90.9 | 6.11 | 0.538 |
| P50502 | ST13 | 4.6 | 1 | 1 | 1 | 369 | 41.3 | 5.27 | 0.699 |
| P31150 | GDI1 | 4.7 | 2 | 4 | 1 | 447 | 50.6 | 5.14 | 0.544 |
| P00748 | F12 | 33.0 | 17 | 77 | 17 | 615 | 67.7 | 7.74 | 0.603 |
| Q14393 | GAS6 | 2.5 | 2 | 3 | 2 | 721 | 79.6 | 6.21 | 0.530 |

| | | | | | | | | | |
|---|---|---|---|---|---|---|---|---|---|
| P13647 | KRT5 | 10.3 | 6 | 12 | 1 | 590 | 62.3 | 7.74 | 0.654 |
| P36980 | CFHR2 | 44.1 | 10 | 42 | 5 | 270 | 30.6 | 6.38 | 0.831 |
| P11279 | LAMP1 | 4.8 | 2 | 5 | 2 | 417 | 44.9 | 8.75 | 0.295 |
| O95897 | OLFM2 | 3.3 | 1 | 1 | 1 | 454 | 51.4 | 7.94 | 0.669 |
| O75636 | FCN3 | 51.5 | 12 | 58 | 12 | 299 | 32.9 | 6.67 | 0.847 |
| Q6UVK1 | CSPG4 | 2.0 | 3 | 4 | 3 | 2322 | 250.4 | 5.47 | 0.653 |
| P05109 | S100A8 | 31.2 | 3 | 6 | 3 | 93 | 10.8 | 7.03 | 0.862 |
| P04275 | VWF | 25.3 | 51 | 109 | 51 | 2813 | 309.1 | 5.48 | 0.728 |
| Q15828 | CST6 | 14.1 | 2 | 4 | 2 | 149 | 16.5 | 8.09 | 0.706 |
| O75368 | SH3BGRL | 10.5 | 1 | 1 | 1 | 114 | 12.8 | 5.25 | 0.790 |
| O60279 | SUSD5 | 4.1 | 2 | 2 | 2 | 629 | 68.0 | 4.91 | 0.847 |
| P37802 | TAGLN2 | 37.7 | 6 | 8 | 6 | 199 | 22.4 | 8.25 | 0.718 |
| Q7Z7M0 | MEGF8 | 2.2 | 4 | 4 | 4 | 2845 | 302.9 | 6.87 | 0.657 |
| P13671 | C6 | 51.3 | 42 | 254 | 42 | 934 | 104.7 | 6.76 | 0.699 |
| P08294 | SOD3 | 30.4 | 5 | 15 | 5 | 240 | 25.8 | 6.61 | 0.743 |
| P06753 | TPM3 | 24.6 | 9 | 16 | 4 | 285 | 32.9 | 4.72 | 0.862 |
| P04066 | FUCA1 | 8.6 | 4 | 5 | 4 | 466 | 53.7 | 6.84 | 0.466 |
| Q12864 | CDH17 | 6.6 | 4 | 4 | 4 | 832 | 92.2 | 5.14 | 0.841 |
| Q14118 | DAG1 | 12.6 | 6 | 14 | 6 | 895 | 97.4 | 8.56 | 0.268 |
| Q14574 | DSC3 | 1.9 | 2 | 2 | 2 | 896 | 99.9 | 6.1 | 0.575 |
| Q86UN3 | RTN4RL2 | 5.2 | 2 | 2 | 2 | 420 | 46.1 | 7.62 | 0.494 |
| P07225 | PROS1 | 37.4 | 20 | 102 | 20 | 676 | 75.1 | 5.67 | 0.456 |
| P20933 | AGA | 3.2 | 1 | 2 | 1 | 346 | 37.2 | 6.28 | 0.663 |
| P06732 | CKM | 4.5 | 1 | 1 | 1 | 381 | 43.1 | 7.25 | 0.215 |
| Q8TDY8 | IGDCC4 | 1.3 | 1 | 1 | 1 | 1250 | 134.1 | 6.2 | 0.669 |
| Q9UBG0 | MRC2 | 6.9 | 6 | 8 | 6 | 1479 | 166.6 | 5.83 | 0.616 |
| Q9Y4L1 | HYOU1 | 10.3 | 7 | 14 | 7 | 999 | 111.3 | 5.22 | 0.470 |
| P54802 | NAGLU | 12.0 | 6 | 6 | 6 | 743 | 82.2 | 6.65 | 0.418 |
| P02753 | RBP4 | 73.1 | 12 | 412 | 12 | 201 | 23.0 | 6.07 | 0.753 |
| P19827 | ITIH1 | 36.2 | 27 | 545 | 27 | 911 | 101.3 | 6.79 | 0.717 |
| Q04756 | HGFAC | 22.3 | 12 | 46 | 12 | 655 | 70.6 | 7.24 | 0.659 |
| P08238 | HSP90AB1 | 10.5 | 6 | 12 | 1 | 724 | 83.2 | 5.03 | 0.386 |
| P05186 | ALPL | 2.1 | 1 | 1 | 1 | 524 | 57.3 | 6.67 | 0.848 |
| Q96RD9 | FCRL5 | 1.7 | 1 | 1 | 1 | 977 | 106.4 | 7.14 | 0.454 |
| Q03591 | CFHR1 | 49.7 | 13 | 87 | 1 | 330 | 37.6 | 7.39 | 0.780 |
| Q9NR34 | MAN1C1 | 1.3 | 1 | 1 | 1 | 630 | 70.9 | 7.46 | 0.750 |
| O00462 | MANBA | 3.4 | 2 | 2 | 2 | 879 | 100.8 | 5.52 | 0.793 |
| Q9NZD8 | SPG21 | 3.2 | 1 | 3 | 1 | 308 | 34.9 | 6.28 | 0.633 |
| P05155 | SERPING1 | 29.2 | 16 | 174 | 16 | 500 | 55.1 | 6.55 | 0.806 |
| P02671 | FGA | 41.0 | 33 | 387 | 33 | 866 | 94.9 | 6.01 | 0.880 |
| P08581 | MET | 3.4 | 3 | 3 | 3 | 1390 | 155.4 | 7.33 | 0.400 |
| Q99972 | MYOC | 6.5 | 3 | 3 | 3 | 504 | 56.9 | 5.54 | 0.779 |
| P48740 | MASP1 | 27.8 | 15 | 38 | 14 | 699 | 79.2 | 5.49 | 0.859 |

| | | | | | | | | | |
|---|---|---|---|---|---|---|---|---|---|
| P27487 | DPP4 | 8.9 | 7 | 14 | 7 | 766 | 88.2 | 6.04 | 0.313 |
| P07108 | DBI | 9.2 | 1 | 1 | 1 | 87 | 10.0 | 6.57 | 0.886 |
| Q6UX71 | PLXDC2 | 14.4 | 6 | 9 | 6 | 529 | 59.5 | 6.46 | 0.374 |
| P52799 | EFNB2 | 4.8 | 1 | 2 | 1 | 333 | 36.9 | 8.85 | 0.779 |
| P15144 | ANPEP | 26.7 | 22 | 40 | 22 | 967 | 109.5 | 5.48 | 0.602 |
| P05156 | CFI | 42.2 | 23 | 249 | 23 | 583 | 65.7 | 7.5 | 0.696 |
| P62937 | PPIA | 35.8 | 5 | 9 | 5 | 165 | 18.0 | 7.81 | 0.804 |
| P12955 | PEPD | 26.4 | 10 | 18 | 10 | 493 | 54.5 | 6 | 0.737 |
| P10153 | RNASE2 | 19.9 | 2 | 7 | 2 | 161 | 18.3 | 8.73 | 0.669 |
| A0A0J9YXX1 | IGHV5-10-1 | 26.5 | 3 | 5 | 1 | 117 | 12.8 | 8.28 | 0.897 |
| Q10588 | BST1 | 20.1 | 6 | 17 | 6 | 318 | 35.7 | 7.8 | 0.790 |
| Q13308 | PTK7 | 1.8 | 2 | 3 | 2 | 1070 | 118.3 | 7.09 | 0.770 |
| P07737 | PFN1 | 65.0 | 6 | 11 | 6 | 140 | 15.0 | 8.27 | 0.889 |
| Q9HCU0 | CD248 | 9.5 | 5 | 7 | 5 | 757 | 80.8 | 5.35 | 0.869 |
| P15169 | CPN1 | 30.3 | 10 | 50 | 10 | 458 | 52.3 | 7.34 | 0.669 |
| P07711 | CTSL | 3.6 | 1 | 3 | 1 | 333 | 37.5 | 5.45 | 0.657 |
| P18428 | LBP | 10.0 | 4 | 10 | 4 | 481 | 53.4 | 6.7 | 0.860 |
| P35590 | TIE1 | 4.0 | 4 | 6 | 4 | 1138 | 125.0 | 7.03 | 0.583 |
| A0A0C4DH25 | IGKV3D-20 | 13.8 | 2 | 3 | 1 | 116 | 12.5 | 4.59 | 0.821 |
| P68104 | EEF1A1 | 8.7 | 3 | 3 | 3 | 462 | 50.1 | 9.01 | 0.924 |
| P49747 | COMP | 26.8 | 15 | 41 | 13 | 757 | 82.8 | 4.6 | 0.889 |
| Q96RW7 | HMCN1 | 0.9 | 4 | 6 | 4 | 5635 | 613.0 | 6.49 | 0.727 |
| P12111 | COL6A3 | 28.7 | 71 | 212 | 71 | 3177 | 343.5 | 6.68 | 0.798 |
| O00187 | MASP2 | 10.3 | 5 | 13 | 5 | 686 | 75.7 | 5.63 | 0.822 |
| P18206 | VCL | 41.4 | 42 | 55 | 42 | 1134 | 123.7 | 5.66 | 0.904 |
| P0DOX3 | | 24.6 | 8 | 29 | 8 | 512 | 56.2 | 8.02 | 0.832 |
| P29279 | CTGF | 3.7 | 1 | 2 | 1 | 349 | 38.1 | 8 | 0.862 |
| P01011 | SERPINA3 | 54.1 | 21 | 1041 | 21 | 423 | 47.6 | 5.52 | 0.705 |
| Q15904 | ATP6AP1 | 3.8 | 1 | 1 | 1 | 470 | 52.0 | 6.14 | 0.776 |
| P61204 | ARF3 | 5.5 | 1 | 1 | 1 | 181 | 20.6 | 7.43 | 0.876 |
| P02144 | MB | 14.3 | 2 | 2 | 2 | 154 | 17.2 | 7.68 | 0.715 |
| P00747 | PLG | 60.4 | 44 | 512 | 44 | 810 | 90.5 | 7.24 | 0.932 |
| P62328 | TMSB4X | 40.9 | 2 | 2 | 2 | 44 | 5.1 | 5.06 | 0.924 |
| P04070 | PROC | 19.1 | 6 | 14 | 6 | 461 | 52.0 | 6.28 | 0.900 |
| Q16394 | EXT1 | 5.4 | 3 | 3 | 3 | 746 | 86.2 | 9.04 | 0.896 |
| Q96B86 | RGMA | 4.0 | 1 | 1 | 1 | 450 | 49.3 | 7.42 | 0.794 |
| P32942 | ICAM3 | 3.3 | 2 | 2 | 2 | 547 | 59.5 | 5.43 | 0.755 |
| Q49AG3 | ZBED5 | 1.0 | 1 | 1 | 1 | 693 | 78.9 | 8.27 | 0.639 |
| P43251 | BTD | 31.9 | 15 | 74 | 15 | 543 | 61.1 | 6.25 | 0.873 |
| Q12905 | ILF2 | 4.1 | 1 | 1 | 1 | 390 | 43.0 | 5.26 | 0.691 |
| O43505 | B4GAT1 | 7.2 | 2 | 3 | 2 | 415 | 47.1 | 7.2 | 0.796 |
| P26927 | MST1 | 43.6 | 23 | 66 | 23 | 711 | 80.3 | 7.68 | 0.891 |
| P04180 | LCAT | 28.4 | 8 | 26 | 8 | 440 | 49.5 | 6.11 | 0.871 |

| | | | | | | | | | |
|---|---|---|---|---|---|---|---|---|---|
| Q9UNN8 | PROCR | 16.4 | 3 | 8 | 3 | 238 | 26.7 | 7.18 | 0.916 |
| P13797 | PLS3 | 10.3 | 6 | 19 | 1 | 630 | 70.8 | 5.6 | 0.888 |
| Q9Y6R7 | FCGBP | 14.4 | 35 | 78 | 35 | 5405 | 571.6 | 5.34 | 0.914 |
| P12110 | COL6A2 | 8.2 | 7 | 10 | 7 | 1019 | 108.5 | 6.21 | 0.789 |
| Q9BUN1 | MENT | 2.3 | 1 | 1 | 1 | 341 | 36.7 | 8.59 | 0.893 |
| P49641 | MAN2A2 | 1.6 | 2 | 2 | 1 | 1150 | 130.5 | 6.84 | 0.834 |
| P20851 | C4BPB | 30.2 | 6 | 21 | 6 | 252 | 28.3 | 5.14 | 0.940 |
| P54289 | CACNA2D1 | 14.3 | 12 | 14 | 12 | 1103 | 124.5 | 5.27 | 0.775 |
| A0A0C4DH38 | IGHV5-51 | 34.2 | 3 | 8 | 1 | 117 | 12.7 | 8.27 | 0.797 |
| Q86SQ4 | ADGRG6 | 0.9 | 1 | 1 | 1 | 1221 | 136.6 | 7.87 | 0.807 |
| Q99983 | OMD | 9.7 | 3 | 4 | 3 | 421 | 49.5 | 5.59 | 0.934 |
| P04003 | C4BPA | 39.4 | 21 | 152 | 21 | 597 | 67.0 | 7.3 | 0.951 |
| P29401 | TKT | 10.8 | 5 | 8 | 5 | 623 | 67.8 | 7.66 | 0.957 |
| A0A0C4DH34 | IGHV4-28 | 7.7 | 1 | 3 | 1 | 117 | 13.1 | 9.29 | 0.961 |
| Q92820 | GGH | 24.8 | 9 | 23 | 9 | 318 | 35.9 | 7.11 | 0.916 |
| P05121 | SERPINE1 | 8.7 | 4 | 4 | 4 | 402 | 45.0 | 7.2 | 0.958 |
| P01344 | IGF2 | 13.9 | 2 | 12 | 2 | 180 | 20.1 | 9.32 | 0.909 |
| Q13740 | ALCAM | 19.7 | 9 | 25 | 9 | 583 | 65.1 | 6.25 | 0.877 |
| P05556 | ITGB1 | 13.0 | 8 | 10 | 8 | 798 | 88.4 | 5.39 | 0.929 |
| P0DOY2 | IGLC2 | 69.8 | 6 | 120 | 3 | 106 | 11.3 | 7.24 | 0.949 |
| Q8NBP7 | PCSK9 | 3.0 | 2 | 2 | 2 | 692 | 74.2 | 6.61 | 0.919 |
| P24821 | TNC | 25.2 | 42 | 95 | 42 | 2201 | 240.7 | 4.89 | 0.969 |
| P23142 | FBLN1 | 27.9 | 15 | 49 | 15 | 703 | 77.2 | 5.22 | 0.966 |
| Q6YHK3 | CD109 | 10.5 | 12 | 20 | 12 | 1445 | 161.6 | 5.85 | 0.954 |
| Q13332 | PTPRS | 2.9 | 3 | 3 | 3 | 1948 | 216.9 | 6.46 | 0.915 |
| P05451 | REG1A | 31.9 | 6 | 27 | 5 | 166 | 18.7 | 5.94 | 0.986 |
| P22570 | FDXR | 4.5 | 1 | 1 | 1 | 491 | 53.8 | 8.44 | 0.965 |
| Q6FHJ7 | SFRP4 | 26.0 | 7 | 16 | 7 | 346 | 39.8 | 8.82 | 0.957 |
| P14625 | HSP90B1 | 21.0 | 16 | 35 | 15 | 803 | 92.4 | 4.84 | 0.945 |
| P07478 | PRSS2 | 15.8 | 3 | 12 | 3 | 247 | 26.5 | 4.92 | 0.989 |
| Q04917 | YWHAH | 22.4 | 6 | 11 | 2 | 246 | 28.2 | 4.84 | 0.983 |
| P86452 | ZBED6 | 1.0 | 1 | 1 | 1 | 979 | 109.9 | 6.55 | 0.903 |
| P02774 | GC | 83.8 | 43 | 1424 | 43 | 474 | 52.9 | 5.54 | 0.981 |
| P06396 | GSN | 66.8 | 39 | 418 | 39 | 782 | 85.6 | 6.28 | 0.985 |
| Q12841 | FSTL1 | 44.8 | 14 | 36 | 14 | 308 | 35.0 | 5.52 | 0.988 |
| Q9BUD6 | SPON2 | 12.4 | 3 | 5 | 3 | 331 | 35.8 | 5.52 | 0.997 |
| A0A075B6K4 | IGLV3-10 | 15.7 | 2 | 4 | 2 | 115 | 12.4 | 4.83 | 0.996 |
| P56715 | RP1 | 0.5 | 1 | 1 | 1 | 2156 | 240.5 | 5.8 | 0.997 |
| P08195 | SLC3A2 | 16.7 | 8 | 12 | 8 | 630 | 68.0 | 5.01 | 1.000 |
| P55268 | LAMB2 | 1.6 | 2 | 4 | 2 | 1798 | 195.9 | 6.52 | 1.000 |
| P00751 | CFB | 58.5 | 42 | 619 | 42 | 764 | 85.5 | 7.06 | 0.993 |
| Q16610 | ECM1 | 51.9 | 22 | 130 | 22 | 540 | 60.6 | 6.71 | 0.990 |
| P05019 | IGF1 | 7.2 | 1 | 2 | 1 | 195 | 21.8 | 9.72 | 0.988 |

| | | | | | | | | | |
|---|---|---|---|---|---|---|---|---|---|
| P21333 | FLNA | 4.6 | 7 | 10 | 7 | 2647 | 280.6 | 6.06 | 0.994 |
| O00339 | MATN2 | 1.2 | 1 | 1 | 1 | 956 | 106.8 | 6.27 | 0.988 |
| Q14515 | SPARCL1 | 35.4 | 20 | 54 | 20 | 664 | 75.2 | 4.81 | 0.975 |
| P01593 | IGKV1D-33 | 13.7 | 1 | 4 | 1 | 117 | 12.8 | 4.78 | 0.950 |
| P04217 | A1BG | 53.3 | 15 | 328 | 15 | 495 | 54.2 | 5.86 | 0.951 |
| Q14019 | COTL1 | 12.0 | 2 | 4 | 2 | 142 | 15.9 | 5.67 | 0.982 |
| Q13449 | LSAMP | 10.1 | 3 | 3 | 3 | 338 | 37.4 | 6.98 | 0.966 |
| Q92859 | NEO1 | 9.5 | 10 | 17 | 10 | 1461 | 159.9 | 6.54 | 0.901 |
| P05062 | ALDOB | 20.6 | 6 | 7 | 5 | 364 | 39.4 | 7.87 | 0.979 |
| P26572 | MGAT1 | 2.5 | 1 | 1 | 1 | 445 | 50.8 | 9.16 | 0.959 |
| P00533 | EGFR | 3.1 | 3 | 3 | 3 | 1210 | 134.2 | 6.68 | 0.883 |
| Q9HCL0 | PCDH18 | 1.7 | 2 | 2 | 2 | 1135 | 126.1 | 5.15 | 0.912 |
| Q14964 | RAB39A | 3.7 | 1 | 1 | 1 | 217 | 25.0 | 7.65 | 0.878 |
| Q9Y4D7 | PLXND1 | 2.2 | 2 | 2 | 2 | 1925 | 211.9 | 7.15 | 0.947 |
| P78417 | GSTO1 | 27.0 | 7 | 10 | 7 | 241 | 27.5 | 6.6 | 0.942 |
| P61916 | NPC2 | 43.0 | 5 | 13 | 5 | 151 | 16.6 | 7.65 | 0.935 |
| P01833 | PIGR | 13.0 | 8 | 12 | 8 | 764 | 83.2 | 5.74 | 0.956 |
| P02766 | TTR | 40.8 | 4 | 15 | 4 | 147 | 15.9 | 5.76 | 0.797 |
| P01033 | TIMP1 | 45.4 | 7 | 47 | 7 | 207 | 23.2 | 8.1 | 0.895 |
| P09668 | CTSH | 12.5 | 3 | 3 | 3 | 335 | 37.4 | 8.07 | 0.747 |
| P01714 | IGLV3-19 | 8.0 | 1 | 1 | 1 | 112 | 12.0 | 4.96 | 0.947 |
| P61254 | RPL26 | 7.6 | 1 | 5 | 1 | 145 | 17.2 | 10.6 | 0.954 |
| P08185 | SERPINA6 | 31.6 | 10 | 90 | 10 | 405 | 45.1 | 6.04 | 0.857 |
| Q8IUX7 | AEBP1 | 6.6 | 7 | 9 | 7 | 1158 | 130.8 | 5.11 | 0.885 |
| Q9NTU7 | CBLN4 | 3.5 | 1 | 2 | 1 | 201 | 21.8 | 8.73 | 0.926 |
| Q07954 | LRP1 | 4.9 | 21 | 31 | 21 | 4544 | 504.3 | 5.39 | 0.886 |
| P10586 | PTPRF | 2.0 | 3 | 3 | 3 | 1907 | 212.7 | 6.3 | 0.833 |
| P0DOX2 | | 38.0 | 10 | 80 | 4 | 455 | 48.9 | 6.67 | 0.956 |
| Q8N5G2 | MACO1 | 1.1 | 1 | 1 | 1 | 664 | 76.1 | 9.07 | 0.887 |
| P08603 | CFH | 57.4 | 62 | 1071 | 54 | 1231 | 139.0 | 6.61 | 0.797 |
| Q9NPY3 | CD93 | 12.1 | 7 | 14 | 7 | 652 | 68.5 | 5.44 | 0.893 |
| A0A0C4DH31 | IGHV1-18 | 9.4 | 1 | 4 | 1 | 117 | 12.8 | 8.84 | 0.936 |
| P07911 | UMOD | 1.4 | 1 | 1 | 1 | 640 | 69.7 | 5.24 | 0.779 |
| Q8NFD2 | ANKK1 | 2.1 | 1 | 1 | 1 | 765 | 84.6 | 7.08 | 0.963 |
| P35442 | THBS2 | 9.8 | 9 | 16 | 9 | 1172 | 129.9 | 4.83 | 0.891 |
| Q504Y2 | PKDCC | 2.0 | 1 | 1 | 1 | 493 | 54.1 | 8.41 | 0.919 |
| O95445 | APOM | 48.4 | 8 | 23 | 8 | 188 | 21.2 | 6.01 | 0.965 |
| P61158 | ACTR3 | 1.9 | 1 | 2 | 1 | 418 | 47.3 | 5.88 | 0.892 |
| P08709 | F7 | 4.1 | 1 | 1 | 1 | 466 | 51.6 | 7.23 | 0.777 |
| Q9H7P6 | MVB12B | 3.4 | 1 | 1 | 1 | 319 | 35.6 | 8.15 | 0.738 |
| P0C0L5 | C4B | 62.2 | 87 | 1469 | 4 | 1744 | 192.6 | 7.27 | 0.892 |
| Q9HBB8 | CDHR5 | 7.1 | 4 | 5 | 4 | 845 | 88.2 | 4.93 | 0.776 |
| Q96HD1 | CRELD1 | 4.5 | 2 | 3 | 2 | 420 | 45.4 | 4.87 | 0.807 |

| | | | | | | | | | |
|---|---|---|---|---|---|---|---|---|---|
| Q14112 | NID2 | 6.5 | 8 | 14 | 8 | 1375 | 151.2 | 5.29 | 0.925 |
| Q6PCE3 | PGM2L1 | 3.9 | 1 | 1 | 1 | 622 | 70.4 | 7.15 | 0.871 |
| Q6PCB0 | VWA1 | 1.6 | 1 | 3 | 1 | 445 | 46.8 | 7.68 | 0.756 |
| P12318 | FCGR2A | 9.8 | 2 | 4 | 2 | 317 | 35.0 | 6.68 | 0.812 |
| P19021 | PAM | 6.9 | 6 | 9 | 6 | 973 | 108.3 | 6.42 | 0.563 |
| P35908 | KRT2 | 23.2 | 14 | 26 | 11 | 639 | 65.4 | 8 | 0.891 |
| P49908 | SELENOP | 12.6 | 6 | 23 | 6 | 381 | 43.2 | 7.87 | 0.872 |
| Q9UIB8 | CD84 | 4.6 | 1 | 1 | 1 | 345 | 38.8 | 7.06 | 0.729 |
| Q93070 | ART4 | 2.9 | 1 | 1 | 1 | 314 | 35.9 | 9.22 | 0.810 |
| P08571 | CD14 | 37.3 | 13 | 76 | 13 | 375 | 40.1 | 6.23 | 0.664 |
| O75144 | ICOSLG | 17.9 | 4 | 6 | 4 | 302 | 33.3 | 5.31 | 0.802 |
| P08572 | COL4A2 | 1.5 | 2 | 3 | 2 | 1712 | 167.4 | 8.66 | 0.867 |
| P0C0L4 | C4A | 61.4 | 86 | 1448 | 3 | 1744 | 192.7 | 7.08 | 0.921 |
| P22105 | TNXB | 16.7 | 41 | 98 | 41 | 4242 | 457.9 | 5.17 | 0.662 |
| Q96IY4 | CPB2 | 26.2 | 11 | 51 | 11 | 423 | 48.4 | 7.71 | 0.906 |
| Q13508 | ART3 | 8.7 | 3 | 4 | 3 | 389 | 43.9 | 6.06 | 0.782 |
| P01701 | IGLV1-51 | 6.8 | 1 | 2 | 1 | 117 | 12.2 | 7.03 | 0.833 |
| P05160 | F13B | 45.7 | 22 | 107 | 22 | 661 | 75.5 | 6.39 | 0.735 |
| Q9P232 | CNTN3 | 1.3 | 1 | 1 | 1 | 1028 | 112.8 | 6.3 | 0.848 |
| O43493 | TGOLN2 | 11.5 | 5 | 6 | 5 | 479 | 51.0 | 5.73 | 0.834 |
| Q16706 | MAN2A1 | 5.4 | 6 | 7 | 5 | 1144 | 131.1 | 7.58 | 0.637 |
| P09601 | HMOX1 | 5.6 | 1 | 1 | 1 | 288 | 32.8 | 8.25 | 0.904 |
| O60462 | NRP2 | 4.1 | 3 | 4 | 3 | 931 | 104.8 | 5.17 | 0.682 |
| O95980 | RECK | 2.1 | 2 | 3 | 2 | 971 | 106.4 | 6.74 | 0.807 |
| Q9BS26 | ERP44 | 5.9 | 2 | 2 | 2 | 406 | 46.9 | 5.26 | 0.533 |
| P06312 | IGKV4-1 | 22.3 | 2 | 6 | 2 | 121 | 13.4 | 5.25 | 0.844 |
| O00391 | QSOX1 | 30.7 | 26 | 75 | 26 | 747 | 82.5 | 8.92 | 0.631 |
| P13497 | BMP1 | 1.6 | 1 | 1 | 1 | 986 | 111.2 | 6.9 | 0.787 |
| P22352 | GPX3 | 35.4 | 7 | 67 | 7 | 226 | 25.5 | 8.13 | 0.844 |
| Q05707 | COL14A1 | 2.1 | 3 | 3 | 3 | 1796 | 193.4 | 5.3 | 0.530 |
| Q93063 | EXT2 | 1.3 | 1 | 3 | 1 | 718 | 82.2 | 6.55 | 0.795 |
| P35555 | FBN1 | 4.9 | 11 | 11 | 11 | 2871 | 312.0 | 4.93 | 0.667 |
| P07359 | GP1BA | 13.3 | 8 | 24 | 8 | 652 | 71.5 | 6.29 | 0.647 |
| O75882 | ATRN | 24.3 | 30 | 117 | 30 | 1429 | 158.4 | 7.31 | 0.584 |
| Q8NHW5 | RPLP0P6 | 2.5 | 1 | 2 | 1 | 317 | 34.3 | 5.55 | 0.796 |
| P17813 | ENG | 9.9 | 6 | 11 | 6 | 658 | 70.5 | 6.61 | 0.710 |
| P15509 | CSF2RA | 3.5 | 1 | 1 | 1 | 400 | 46.2 | 7.75 | 0.834 |
| P28838 | LAP3 | 7.1 | 2 | 2 | 2 | 519 | 56.1 | 7.93 | 0.781 |
| P36955 | SERPINF1 | 54.5 | 21 | 191 | 21 | 418 | 46.3 | 6.38 | 0.814 |
| Q9Y279 | VSIG4 | 20.1 | 6 | 12 | 6 | 399 | 44.0 | 6.35 | 0.760 |
| P01023 | A2M | 54.3 | 62 | 848 | 54 | 1474 | 163.2 | 6.46 | 0.776 |
| P04264 | KRT1 | 27.3 | 19 | 62 | 18 | 644 | 66.0 | 8.12 | 0.899 |
| Q5SYB0 | FRMPD1 | 0.4 | 1 | 2 | 1 | 1578 | 173.3 | 5.25 | 0.871 |

| | | | | | | | | | |
|---|---|---|---|---|---|---|---|---|---|
| P02647 | APOA1 | 50.6 | 14 | 52 | 14 | 267 | 30.8 | 5.76 | 0.691 |
| Q9BY67 | CADM1 | 9.3 | 3 | 5 | 3 | 442 | 48.5 | 5.07 | 0.075 |
| P23470 | PTPRG | 4.3 | 5 | 8 | 5 | 1445 | 161.9 | 6.42 | 0.698 |
| Q7Z7M8 | B3GNT8 | 2.8 | 1 | 1 | 1 | 397 | 43.4 | 8.32 | 0.300 |
| Q9H8J5 | MANSC1 | 3.5 | 1 | 1 | 1 | 431 | 46.8 | 6.54 | 0.803 |
| P02760 | AMBP | 47.7 | 14 | 270 | 14 | 352 | 39.0 | 6.25 | 0.542 |
| P30043 | BLVRB | 7.3 | 1 | 1 | 1 | 206 | 22.1 | 7.65 | 0.886 |
| Q9H8L6 | MMRN2 | 4.7 | 3 | 8 | 3 | 949 | 104.3 | 5.86 | 0.673 |
| Q9H4G4 | GLIPR2 | 11.0 | 1 | 1 | 1 | 154 | 17.2 | 9.41 | 0.703 |
| P06681 | C2 | 45.1 | 30 | 251 | 30 | 752 | 83.2 | 7.42 | 0.696 |
| P35443 | THBS4 | 31.6 | 20 | 56 | 17 | 961 | 105.8 | 4.68 | 0.798 |
| P52566 | ARHGDIB | 6.5 | 1 | 1 | 1 | 201 | 23.0 | 5.21 | 0.781 |
| O15031 | PLXNB2 | 1.1 | 2 | 2 | 2 | 1838 | 205.0 | 6.24 | 0.610 |
| P07357 | C8A | 52.6 | 22 | 244 | 22 | 584 | 65.1 | 6.47 | 0.421 |
| P31146 | CORO1A | 1.7 | 1 | 1 | 1 | 461 | 51.0 | 6.68 | 0.632 |
| Q14520 | HABP2 | 28.9 | 13 | 99 | 13 | 560 | 62.6 | 6.54 | 0.491 |
| P07360 | C8G | 58.4 | 11 | 82 | 11 | 202 | 22.3 | 8.31 | 0.294 |
| Q7Z7G0 | ABI3BP | 13.9 | 11 | 26 | 11 | 1075 | 118.6 | 9.44 | 0.742 |
| P07998 | RNASE1 | 14.7 | 1 | 3 | 1 | 156 | 17.6 | 8.79 | 0.823 |
| Q16853 | AOC3 | 10.5 | 7 | 11 | 7 | 763 | 84.6 | 6.52 | 0.397 |
| Q99969 | RARRES2 | 6.7 | 1 | 1 | 1 | 163 | 18.6 | 9.09 | 0.547 |
| A0A075B6I0 | IGLV8-61 | 7.4 | 1 | 4 | 1 | 122 | 12.8 | 4.55 | 0.866 |
| P28062 | PSMB8 | 4.3 | 1 | 1 | 1 | 276 | 30.3 | 7.43 | 0.581 |
| Q8TDQ0 | HAVCR2 | 8.3 | 2 | 2 | 2 | 301 | 33.4 | 5.72 | 0.720 |
| P35527 | KRT9 | 31.6 | 16 | 30 | 15 | 623 | 62.0 | 5.24 | 0.883 |
| P19823 | ITIH2 | 53.2 | 38 | 578 | 38 | 946 | 106.4 | 6.86 | 0.810 |
| Q15848 | ADIPOQ | 27.0 | 5 | 16 | 5 | 244 | 26.4 | 5.74 | 0.722 |
| Q86UD1 | OAF | 32.2 | 6 | 20 | 6 | 273 | 30.7 | 6.84 | 0.676 |
| Q9UNW1 | MINPP1 | 26.7 | 11 | 18 | 11 | 487 | 55.0 | 7.81 | 0.305 |
| P10721 | KIT | 2.8 | 3 | 5 | 3 | 976 | 109.8 | 6.98 | 0.569 |
| P00488 | F13A1 | 26.9 | 18 | 61 | 18 | 732 | 83.2 | 6.09 | 0.668 |
| P25311 | AZGP1 | 58.1 | 20 | 336 | 20 | 298 | 34.2 | 6.05 | 0.635 |
| Q9UBP4 | DKK3 | 26.3 | 6 | 18 | 6 | 350 | 38.4 | 4.65 | 0.412 |
| P07686 | HEXB | 4.7 | 3 | 4 | 3 | 556 | 63.1 | 6.76 | 0.585 |
| Q9GZX9 | TWSG1 | 4.0 | 1 | 1 | 1 | 223 | 25.0 | 5.34 | 0.569 |
| Q01459 | CTBS | 18.7 | 6 | 13 | 6 | 385 | 43.7 | 6.64 | 0.658 |
| P30530 | AXL | 2.0 | 1 | 1 | 1 | 894 | 98.3 | 5.39 | 0.655 |
| P01031 | C5 | 58.8 | 81 | 618 | 81 | 1676 | 188.2 | 6.52 | 0.402 |
| Q92484 | SMPDL3A | 2.0 | 1 | 1 | 1 | 453 | 51.2 | 6.33 | 0.378 |
| Q16787 | LAMA3 | 0.5 | 1 | 1 | 1 | 3333 | 366.4 | 7.24 | 0.727 |
| Q8TER0 | SNED1 | 1.0 | 1 | 2 | 1 | 1413 | 152.1 | 6.98 | 0.636 |
| P33151 | CDH5 | 15.3 | 11 | 34 | 11 | 784 | 87.5 | 5.43 | 0.702 |
| P40189 | IL6ST | 10.5 | 7 | 7 | 7 | 918 | 103.5 | 5.95 | 0.570 |

| | | | | | | | | | |
|---|---|---|---|---|---|---|---|---|---|
| Q8TAT6 | NPLOC4 | 1.2 | 1 | 1 | 1 | 608 | 68.1 | 6.38 | 0.497 |
| P09382 | LGALS1 | 44.4 | 5 | 7 | 5 | 135 | 14.7 | 5.5 | 0.725 |
| Q96AP7 | ESAM | 4.6 | 2 | 3 | 2 | 390 | 41.2 | 9.32 | 0.629 |
| A6QL64 | ANKRD36 | 0.9 | 1 | 1 | 1 | 1941 | 217.3 | 8.66 | 0.584 |
| P04792 | HSPB1 | 9.3 | 2 | 2 | 2 | 205 | 22.8 | 6.4 | 0.858 |
| P07358 | C8B | 50.4 | 28 | 188 | 28 | 591 | 67.0 | 8.13 | 0.113 |
| P13489 | RNH1 | 7.4 | 2 | 2 | 2 | 461 | 49.9 | 4.82 | 0.447 |
| Q9HBR0 | SLC38A10 | 2.9 | 2 | 3 | 2 | 1119 | 119.7 | 5.73 | 0.268 |
| P01619 | IGKV3-20 | 13.8 | 2 | 3 | 1 | 116 | 12.5 | 4.96 | 0.652 |
| P53634 | CTSC | 10.6 | 3 | 5 | 3 | 463 | 51.8 | 6.99 | 0.512 |
| P22692 | IGFBP4 | 34.9 | 7 | 19 | 7 | 258 | 27.9 | 7.15 | 0.608 |
| Q6UY14 | ADAMTSL4 | 3.3 | 3 | 5 | 3 | 1074 | 116.5 | 8.34 | 0.275 |
| A0A0C4DH55 | IGKV3D-7 | 5.9 | 1 | 2 | 1 | 119 | 13.1 | 5.94 | 0.779 |
| Q9BXX0 | EMILIN2 | 1.2 | 1 | 1 | 1 | 1053 | 115.6 | 6.46 | 0.691 |
| Q9UBR2 | CTSZ | 15.8 | 5 | 6 | 5 | 303 | 33.8 | 7.11 | 0.311 |
| Q9BWV1 | BOC | 2.0 | 1 | 2 | 1 | 1114 | 121.0 | 7.01 | 0.766 |
| Q9NZL9 | MAT2B | 5.4 | 1 | 3 | 1 | 334 | 37.5 | 7.36 | 0.818 |
| P13598 | ICAM2 | 20.0 | 5 | 18 | 5 | 275 | 30.6 | 7.43 | 0.610 |
| A0A0B4J1V0 | IGHV3-15 | 5.9 | 1 | 3 | 1 | 119 | 12.9 | 8.62 | 0.518 |
| Q76M96 | CCDC80 | 1.8 | 1 | 1 | 1 | 950 | 108.1 | 9.72 | 0.593 |
| Q9UJJ9 | GNPTG | 23.9 | 5 | 12 | 5 | 305 | 34.0 | 6.95 | 0.456 |
| P09619 | PDGFRB | 2.9 | 2 | 2 | 2 | 1106 | 123.9 | 4.98 | 0.579 |
| Q00532 | CDKL1 | 2.0 | 1 | 8 | 1 | 358 | 41.8 | 8.85 | 0.644 |
| P13645 | KRT10 | 38.4 | 18 | 43 | 16 | 584 | 58.8 | 5.21 | 0.719 |
| P27930 | IL1R2 | 2.0 | 1 | 2 | 1 | 398 | 45.4 | 7.9 | 0.709 |
| P55290 | CDH13 | 17.1 | 10 | 16 | 10 | 713 | 78.2 | 4.98 | 0.433 |
| P15291 | B4GALT1 | 12.8 | 3 | 7 | 3 | 398 | 43.9 | 8.65 | 0.220 |
| O14498 | ISLR | 14.3 | 5 | 11 | 5 | 428 | 46.0 | 5.15 | 0.408 |
| Q9NS71 | GKN1 | 7.0 | 1 | 3 | 1 | 199 | 22.0 | 6.32 | 0.748 |
| Q15223 | NECTIN1 | 9.9 | 3 | 3 | 3 | 517 | 57.1 | 6.1 | 0.465 |
| Q96FE7 | PIK3IP1 | 4.2 | 1 | 1 | 1 | 263 | 28.2 | 5.01 | 0.685 |
| P16070 | CD44 | 5.3 | 4 | 16 | 4 | 742 | 81.5 | 5.33 | 0.601 |
| Q9BTY2 | FUCA2 | 24.2 | 11 | 17 | 11 | 467 | 54.0 | 6.25 | 0.547 |
| Q9NPH3 | IL1RAP | 13.3 | 9 | 24 | 9 | 570 | 65.4 | 8.12 | 0.479 |
| Q12884 | FAP | 2.0 | 2 | 9 | 1 | 760 | 87.7 | 6.65 | 0.609 |
| P11362 | FGFR1 | 5.7 | 4 | 5 | 4 | 822 | 91.8 | 6.21 | 0.635 |
| P13796 | LCP1 | 58.1 | 31 | 101 | 26 | 627 | 70.2 | 5.43 | 0.588 |
| P12821 | ACE | 2.8 | 3 | 3 | 3 | 1306 | 149.6 | 6.39 | 0.103 |
| Q9NY97 | B3GNT2 | 17.1 | 5 | 6 | 5 | 397 | 46.0 | 8.54 | 0.096 |
| P00450 | CP | 67.7 | 60 | 1192 | 60 | 1065 | 122.1 | 5.72 | 0.093 |
| P0DOX8 | | 41.7 | 8 | 110 | 4 | 216 | 22.8 | 6.76 | 0.746 |
| P01782 | IGHV3-9 | 9.3 | 1 | 1 | 1 | 118 | 12.9 | 7.08 | 0.508 |
| Q4KWH8 | PLCH1 | 0.5 | 1 | 2 | 1 | 1693 | 189.1 | 7.74 | 0.500 |

| | | | | | | | | | |
|---|---|---|---|---|---|---|---|---|---|
| P01742 | IGHV1-69 | 15.4 | 2 | 3 | 2 | 117 | 12.7 | 9.17 | 0.786 |
| P48637 | GSS | 5.5 | 3 | 3 | 3 | 474 | 52.4 | 5.92 | 0.227 |
| P19022 | CDH2 | 9.1 | 5 | 8 | 5 | 906 | 99.7 | 4.81 | 0.323 |
| P03951 | F11 | 39.2 | 21 | 66 | 21 | 625 | 70.1 | 8.1 | 0.579 |
| P07333 | CSF1R | 6.7 | 4 | 13 | 4 | 972 | 107.9 | 6.37 | 0.445 |
| P02745 | C1QA | 29.0 | 6 | 21 | 6 | 245 | 26.0 | 9.11 | 0.712 |
| P39060 | COL18A1 | 1.7 | 2 | 2 | 2 | 1754 | 178.1 | 6.01 | 0.497 |
| P21810 | BGN | 3.0 | 1 | 1 | 1 | 368 | 41.6 | 7.52 | 0.691 |
| P07307 | ASGR2 | 16.4 | 5 | 6 | 5 | 311 | 35.1 | 6.25 | 0.374 |
| P02765 | AHSG | 51.0 | 13 | 624 | 13 | 367 | 39.3 | 5.72 | 0.446 |
| P04222 | HLA-C | 6.0 | 2 | 5 | 1 | 366 | 40.8 | 6.39 | 0.804 |
| O15394 | NCAM2 | 7.8 | 5 | 6 | 5 | 837 | 93.0 | 5.6 | 0.256 |
| Q9NPR2 | SEMA4B | 5.0 | 5 | 5 | 5 | 832 | 92.1 | 6.95 | 0.569 |
| O75083 | WDR1 | 1.3 | 1 | 1 | 1 | 606 | 66.2 | 6.65 | 0.462 |
| P05362 | ICAM1 | 18.0 | 7 | 17 | 7 | 532 | 57.8 | 7.99 | 0.449 |
| Q8IWV2 | CNTN4 | 4.2 | 3 | 5 | 3 | 1026 | 113.4 | 7.47 | 0.625 |
| P55103 | INHBC | 2.3 | 1 | 1 | 1 | 352 | 38.2 | 7.11 | 0.704 |
| Q5KU26 | COLEC12 | 0.9 | 1 | 2 | 1 | 742 | 81.5 | 5.69 | 0.391 |
| Q8WZ75 | ROBO4 | 7.1 | 6 | 7 | 6 | 1007 | 107.4 | 6.64 | 0.630 |
| P05413 | FABP3 | 6.8 | 1 | 2 | 1 | 133 | 14.8 | 6.8 | 0.531 |
| Q9GZM5 | YIPF3 | 2.9 | 1 | 1 | 1 | 350 | 38.2 | 5.76 | 0.387 |
| P05452 | CLEC3B | 70.8 | 11 | 88 | 11 | 202 | 22.5 | 5.67 | 0.503 |
| P08637 | FCGR3A | 12.2 | 4 | 18 | 1 | 254 | 29.1 | 8.07 | 0.366 |
| O00533 | CHL1 | 18.0 | 18 | 39 | 18 | 1208 | 135.0 | 5.76 | 0.319 |
| Q9UQP3 | TNN | 1.8 | 1 | 8 | 1 | 1299 | 143.9 | 5.63 | 0.300 |
| P20742 | PZP | 26.1 | 34 | 256 | 26 | 1482 | 163.8 | 6.38 | 0.764 |
| P13591 | NCAM1 | 17.1 | 12 | 31 | 12 | 858 | 94.5 | 4.87 | 0.167 |
| P17900 | GM2A | 45.1 | 7 | 13 | 7 | 193 | 20.8 | 5.31 | 0.390 |
| P01042 | KNG1 | 59.2 | 43 | 742 | 43 | 644 | 71.9 | 6.81 | 0.360 |
| P33908 | MAN1A1 | 17.2 | 10 | 22 | 10 | 653 | 72.9 | 6.47 | 0.600 |
| P12259 | F5 | 6.2 | 11 | 15 | 11 | 2224 | 251.5 | 6.05 | 0.533 |
| Q9NZ08 | ERAP1 | 5.2 | 4 | 5 | 4 | 941 | 107.2 | 6.46 | 0.225 |
| P98095 | FBLN2 | 3.5 | 2 | 2 | 2 | 1184 | 126.5 | 4.82 | 0.474 |
| P02746 | C1QB | 39.1 | 8 | 71 | 8 | 253 | 26.7 | 8.63 | 0.662 |
| P16284 | PECAM1 | 1.9 | 1 | 1 | 1 | 738 | 82.5 | 6.99 | 0.001 |
| P09172 | DBH | 28.2 | 13 | 32 | 13 | 617 | 69.0 | 6.42 | 0.858 |
| Q96S96 | PEBP4 | 20.7 | 4 | 9 | 4 | 227 | 25.7 | 6.54 | 0.635 |
| Q13103 | SPP2 | 10.9 | 2 | 3 | 2 | 211 | 24.3 | 8.32 | 0.562 |
| O14786 | NRP1 | 20.3 | 14 | 28 | 14 | 923 | 103.1 | 5.88 | 0.320 |
| P43121 | MCAM | 19.5 | 9 | 26 | 9 | 646 | 71.6 | 5.76 | 0.390 |
| Q12860 | CNTN1 | 16.7 | 14 | 20 | 14 | 1018 | 113.2 | 5.9 | 0.190 |
| P54108 | CRISP3 | 17.6 | 4 | 12 | 4 | 245 | 27.6 | 7.8 | 0.659 |
| Q99784 | OLFM1 | 11.5 | 5 | 19 | 5 | 485 | 55.3 | 6.95 | 0.073 |

| | | | | | | | | | |
|---|---|---|---|---|---|---|---|---|---|
| Q6EMK4 | VASN | 11.0 | 6 | 21 | 6 | 673 | 71.7 | 7.39 | 0.369 |
| Q14624 | ITIH4 | 56.8 | 41 | 609 | 41 | 930 | 103.3 | 6.98 | 0.247 |
| P09960 | LTA4H | 20.5 | 10 | 16 | 10 | 611 | 69.2 | 6.18 | 0.096 |
| O00241 | SIRPB1 | 5.3 | 2 | 2 | 1 | 398 | 43.2 | 6.52 | 0.627 |
| P20774 | OGN | 40.9 | 13 | 37 | 13 | 298 | 33.9 | 5.63 | 0.705 |
| P78324 | SIRPA | 9.3 | 4 | 4 | 3 | 504 | 54.9 | 6.98 | 0.324 |
| Q08554 | DSC1 | 1.5 | 1 | 1 | 1 | 894 | 99.9 | 5.43 | 0.505 |
| P04196 | HRG | 43.2 | 21 | 379 | 21 | 525 | 59.5 | 7.5 | 0.471 |
| P80108 | GPLD1 | 20.0 | 17 | 52 | 17 | 840 | 92.3 | 6.37 | 0.594 |
| P26992 | CNTFR | 2.4 | 1 | 2 | 1 | 372 | 40.6 | 6.76 | 0.448 |
| P61769 | B2M | 55.5 | 5 | 17 | 5 | 119 | 13.7 | 6.52 | 0.532 |
| Q12913 | PTPRJ | 5.0 | 5 | 9 | 5 | 1337 | 145.9 | 5.58 | 0.317 |
| P09467 | FBP1 | 4.7 | 1 | 2 | 1 | 338 | 36.8 | 6.99 | 0.538 |
| P18065 | IGFBP2 | 34.8 | 9 | 37 | 9 | 325 | 34.8 | 7.5 | 0.489 |
| Q9H6X2 | ANTXR1 | 1.8 | 1 | 1 | 1 | 564 | 62.7 | 7.61 | 0.354 |
| Q02809 | PLOD1 | 2.1 | 1 | 1 | 1 | 727 | 83.5 | 6.95 | 0.546 |
| P07942 | LAMB1 | 22.8 | 32 | 68 | 32 | 1786 | 197.9 | 4.94 | 0.314 |
| P27918 | CFP | 33.0 | 12 | 49 | 12 | 469 | 51.2 | 7.9 | 0.525 |
| O95497 | VNN1 | 16.2 | 6 | 11 | 6 | 513 | 57.0 | 5.55 | 0.712 |
| Q99941 | ATF6B | 1.4 | 1 | 1 | 1 | 703 | 76.7 | 6.27 | 0.231 |
| P01876 | IGHA1 | 56.4 | 14 | 213 | 9 | 353 | 37.6 | 6.51 | 0.662 |
| P01706 | IGLV2-11 | 13.4 | 2 | 2 | 1 | 119 | 12.6 | 7.24 | 0.826 |
| A0A0C4DH68 | IGKV2-24 | 10.8 | 1 | 2 | 1 | 120 | 13.1 | 8.53 | 0.727 |
| P05534 | HLA-A | 18.9 | 6 | 11 | 5 | 365 | 40.7 | 6.34 | 0.821 |
| P10643 | C7 | 64.4 | 42 | 472 | 41 | 843 | 93.5 | 6.48 | 0.489 |
| A0A0B4J1X5 | IGHV3-74 | 15.4 | 2 | 4 | 1 | 117 | 12.8 | 8.66 | 0.437 |
| P24043 | LAMA2 | 14.0 | 37 | 55 | 37 | 3122 | 343.7 | 6.4 | 0.102 |
| P23381 | WARS | 6.8 | 2 | 2 | 2 | 471 | 53.1 | 6.23 | 0.137 |
| Q96EE4 | CCDC126 | 8.6 | 1 | 2 | 1 | 140 | 15.7 | 9.88 | 0.442 |
| Q9ULI3 | HEG1 | 2.6 | 3 | 8 | 3 | 1381 | 147.4 | 6.18 | 0.590 |
| P68032 | ACTC1 | 41.6 | 12 | 63 | 3 | 377 | 42.0 | 5.39 | 0.155 |
| P61160 | ACTR2 | 3.0 | 1 | 1 | 1 | 394 | 44.7 | 6.74 | 0.478 |
| P08174 | CD55 | 8.9 | 4 | 8 | 4 | 381 | 41.4 | 7.59 | 0.104 |
| O60888 | CUTA | 7.8 | 1 | 1 | 1 | 179 | 19.1 | 5.5 | 0.589 |
| Q9H8M5 | CNNM2 | 0.8 | 1 | 1 | 1 | 875 | 96.6 | 6.38 | 0.687 |
| P02750 | LRG1 | 51.6 | 13 | 205 | 13 | 347 | 38.2 | 6.95 | 0.419 |
| P0DOX6 | | 37.8 | 17 | 145 | 1 | 576 | 63.4 | 7.87 | 0.761 |
| Q9NPG4 | PCDH12 | 1.0 | 1 | 1 | 1 | 1184 | 128.9 | 5.29 | 0.523 |
| P15151 | PVR | 9.8 | 4 | 13 | 4 | 417 | 45.3 | 6.52 | 0.122 |
| P27348 | YWHAQ | 22.0 | 6 | 10 | 1 | 245 | 27.7 | 4.78 | 0.770 |
| P0DOX7 | | 53.7 | 7 | 96 | 3 | 214 | 23.4 | 7.17 | 0.473 |
| Q9NZK5 | ADA2 | 17.6 | 8 | 10 | 8 | 511 | 58.9 | 7.91 | 0.169 |
| Q99650 | OSMR | 1.0 | 1 | 1 | 1 | 979 | 110.4 | 5.82 | 0.350 |

| | | | | | | | | | |
|---|---|---|---|---|---|---|---|---|---|
| P14543 | NID1 | 3.8 | 5 | 7 | 5 | 1247 | 136.3 | 5.29 | 0.274 |
| P11047 | LAMC1 | 22.1 | 28 | 49 | 28 | 1609 | 177.5 | 5.12 | 0.303 |
| P35916 | FLT4 | 1.8 | 1 | 1 | 1 | 1363 | 152.7 | 6.3 | 0.738 |
| Q6UXB8 | PI16 | 22.2 | 8 | 29 | 8 | 463 | 49.4 | 5.39 | 0.549 |
| P10909 | CLU | 31.0 | 15 | 153 | 15 | 449 | 52.5 | 6.27 | 0.457 |
| P13987 | CD59 | 23.4 | 3 | 5 | 3 | 128 | 14.2 | 6.48 | 0.255 |
| Q13822 | ENPP2 | 25.4 | 16 | 42 | 16 | 863 | 98.9 | 7.37 | 0.398 |
| Q86VB7 | CD163 | 26.5 | 25 | 89 | 25 | 1156 | 125.4 | 5.95 | 0.491 |
| Q9NQ79 | CRTAC1 | 14.1 | 8 | 18 | 8 | 661 | 71.4 | 5.12 | 0.236 |
| Q9HDC9 | APMAP | 7.2 | 3 | 5 | 3 | 416 | 46.5 | 6.16 | 0.682 |
| Q6KB66 | KRT80 | 1.5 | 1 | 1 | 1 | 452 | 50.5 | 5.67 | 0.681 |
| P02748 | C9 | 52.1 | 27 | 269 | 27 | 559 | 63.1 | 5.59 | 0.082 |
| P14207 | FOLR2 | 23.5 | 5 | 10 | 5 | 255 | 29.3 | 7.53 | 0.398 |
| P04004 | VTN | 43.1 | 14 | 249 | 14 | 478 | 54.3 | 5.8 | 0.536 |
| Q16363 | LAMA4 | 7.1 | 10 | 18 | 10 | 1823 | 202.4 | 6.28 | 0.280 |
| Q9UGM5 | FETUB | 35.1 | 13 | 66 | 13 | 382 | 42.0 | 6.83 | 0.176 |
| Q9BWP8 | COLEC11 | 19.9 | 6 | 15 | 6 | 271 | 28.6 | 5.41 | 0.524 |
| Q6ZN30 | BNC2 | 1.0 | 1 | 8 | 1 | 1099 | 122.3 | 6.52 | 0.527 |
| P02747 | C1QC | 25.3 | 5 | 36 | 5 | 245 | 25.8 | 8.41 | 0.544 |
| O95998 | IL18BP | 8.8 | 2 | 3 | 2 | 194 | 21.1 | 7.39 | 0.380 |
| P51884 | LUM | 43.2 | 14 | 253 | 14 | 338 | 38.4 | 6.61 | 0.365 |
| P01700 | IGLV1-47 | 11.1 | 1 | 1 | 1 | 117 | 12.3 | 5.91 | 0.358 |
| A0A0B4J1U7 | IGHV6-1 | 13.2 | 2 | 6 | 2 | 121 | 13.5 | 9.2 | 0.562 |
| P01008 | SERPINC1 | 58.6 | 31 | 1018 | 31 | 464 | 52.6 | 6.71 | 0.131 |
| P14151 | SELL | 19.4 | 8 | 39 | 8 | 372 | 42.2 | 6.6 | 0.279 |
| P01034 | CST3 | 47.9 | 6 | 47 | 6 | 146 | 15.8 | 8.75 | 0.090 |
| P21583 | KITLG | 3.3 | 1 | 1 | 1 | 273 | 30.9 | 6.14 | 0.295 |
| P15090 | FABP4 | 28.8 | 4 | 6 | 4 | 132 | 14.7 | 7.14 | 0.405 |
| P00742 | F10 | 23.4 | 11 | 20 | 11 | 488 | 54.7 | 5.94 | 0.441 |
| P55058 | PLTP | 30.6 | 14 | 57 | 14 | 493 | 54.7 | 7.01 | 0.336 |
| Q16270 | IGFBP7 | 30.1 | 8 | 21 | 8 | 282 | 29.1 | 7.9 | 0.265 |
| P01834 | IGKC | 80.4 | 6 | 144 | 2 | 107 | 11.8 | 6.52 | 0.598 |
| Q8WUJ3 | CEMIP | 0.7 | 1 | 1 | 1 | 1361 | 152.9 | 7.85 | 0.099 |
| Q12805 | EFEMP1 | 43.6 | 17 | 62 | 17 | 493 | 54.6 | 5.07 | 0.373 |
| Q9Y6Z7 | COLEC10 | 14.8 | 4 | 6 | 4 | 277 | 30.7 | 7.33 | 0.194 |
| P0DJD7 | PGA4 | 2.1 | 1 | 1 | 1 | 388 | 42.0 | 4.34 | 0.402 |
| P09871 | C1S | 41.3 | 23 | 171 | 22 | 688 | 76.6 | 4.96 | 0.455 |
| Q16663 | CCL15 | 16.8 | 1 | 1 | 1 | 113 | 12.2 | 8.18 | 0.092 |
| Q96NZ9 | PRAP1 | 41.1 | 4 | 6 | 4 | 151 | 17.2 | 5.36 | 0.469 |
| P00746 | CFD | 62.8 | 14 | 67 | 14 | 253 | 27.0 | 7.71 | 0.273 |
| Q9GZP0 | PDGFD | 3.2 | 1 | 1 | 1 | 370 | 42.8 | 7.97 | 0.674 |
| O94769 | ECM2 | 5.7 | 3 | 3 | 3 | 699 | 79.7 | 5.41 | 0.232 |
| P01780 | IGHV3-7 | 23.1 | 3 | 11 | 2 | 117 | 12.9 | 6.57 | 0.486 |

| | | | | | | | | | |
|---|---|---|---|---|---|---|---|---|---|
| P05090 | APOD | 30.2 | 6 | 57 | 6 | 189 | 21.3 | 5.15 | 0.391 |
| O43866 | CD5L | 47.8 | 12 | 41 | 12 | 347 | 38.1 | 5.47 | 0.440 |
| Q92520 | FAM3C | 37.4 | 7 | 14 | 7 | 227 | 24.7 | 8.29 | 0.085 |
| Q03167 | TGFBR3 | 5.4 | 3 | 4 | 3 | 851 | 93.4 | 5.71 | 0.104 |
| O75382 | TRIM3 | 1.9 | 1 | 1 | 1 | 744 | 80.8 | 7.83 | 0.100 |
| Q02487 | DSC2 | 6.8 | 4 | 6 | 4 | 901 | 99.9 | 5.34 | 0.213 |
| P19971 | TYMP | 9.3 | 3 | 3 | 3 | 482 | 49.9 | 5.53 | 0.276 |
| P09603 | CSF1 | 4.3 | 2 | 3 | 2 | 554 | 60.1 | 5.29 | 0.165 |
| Q9Y646 | CPQ | 7.2 | 3 | 3 | 3 | 472 | 51.9 | 6.18 | 0.173 |
| P01591 | JCHAIN | 41.5 | 6 | 24 | 6 | 159 | 18.1 | 5.24 | 0.323 |
| P28482 | MAPK1 | 1.9 | 1 | 7 | 1 | 360 | 41.4 | 6.98 | 0.226 |
| P00740 | F9 | 35.4 | 12 | 36 | 12 | 461 | 51.7 | 5.47 | 0.460 |
| P41271 | NBL1 | 7.7 | 2 | 3 | 2 | 181 | 19.4 | 5.29 | 0.132 |
| P01824 | IGHV4-39 | 20.0 | 2 | 9 | 2 | 125 | 13.9 | 9.26 | 0.286 |
| Q9UM47 | NOTCH3 | 0.5 | 1 | 1 | 1 | 2321 | 243.5 | 5.39 | 0.362 |
| Q8N3T6 | TMEM132C | 1.6 | 1 | 1 | 1 | 1108 | 121.7 | 6.35 | 0.525 |
| P22897 | MRC1 | 10.0 | 13 | 23 | 13 | 1456 | 165.9 | 6.54 | 0.275 |
| Q7Z3B1 | NEGR1 | 6.5 | 2 | 6 | 2 | 354 | 38.7 | 6.21 | 0.128 |
| P11684 | SCGB1A1 | 27.5 | 3 | 3 | 3 | 91 | 10.0 | 5.06 | 0.084 |
| Q4LDE5 | SVEP1 | 21.4 | 60 | 130 | 60 | 3571 | 389.9 | 5.5 | 0.379 |
| P00736 | C1R | 48.8 | 26 | 166 | 24 | 705 | 80.1 | 6.21 | 0.446 |
| P19320 | VCAM1 | 34.8 | 22 | 59 | 22 | 739 | 81.2 | 5.22 | 0.235 |
| Q8IZF2 | ADGRF5 | 4.4 | 4 | 5 | 4 | 1346 | 149.4 | 6.65 | 0.058 |
| O76061 | STC2 | 7.0 | 2 | 2 | 2 | 302 | 33.2 | 7.3 | 0.285 |
| Q9UBX1 | CTSF | 4.5 | 2 | 2 | 2 | 484 | 53.3 | 8.22 | 0.219 |
| P24592 | IGFBP6 | 27.9 | 4 | 11 | 4 | 240 | 25.3 | 7.81 | 0.183 |
| Q76LX8 | ADAMTS13 | 8.1 | 10 | 11 | 10 | 1427 | 153.5 | 7.17 | 0.062 |
| O75093 | SLIT1 | 0.6 | 1 | 1 | 1 | 1534 | 167.8 | 6.57 | 0.210 |
| P01871 | IGHM | 62.9 | 21 | 186 | 5 | 453 | 49.4 | 6.77 | 0.548 |
| Q96PD5 | PGLYRP2 | 35.1 | 13 | 176 | 13 | 576 | 62.2 | 7.55 | 0.386 |
| P08887 | IL6R | 1.7 | 1 | 1 | 1 | 468 | 51.5 | 8.22 | 0.159 |
| P08519 | LPA | 19.4 | 5 | 9 | 5 | 4548 | 501.0 | 5.88 | 0.403 |
| P41222 | PTGDS | 25.3 | 5 | 45 | 5 | 190 | 21.0 | 7.8 | 0.375 |
| P04746 | AMY2A | 14.5 | 6 | 12 | 6 | 511 | 57.7 | 7.05 | 0.486 |
| Q06033 | ITIH3 | 30.2 | 22 | 123 | 22 | 890 | 99.8 | 5.74 | 0.215 |
| P23471 | PTPRZ1 | 0.4 | 1 | 1 | 1 | 2315 | 254.4 | 4.88 | 0.220 |
| P05543 | SERPINA7 | 56.9 | 22 | 213 | 22 | 415 | 46.3 | 6.3 | 0.062 |
| Q9UHG3 | PCYOX1 | 10.5 | 5 | 6 | 5 | 505 | 56.6 | 6.18 | 0.293 |
| P16035 | TIMP2 | 35.5 | 9 | 18 | 9 | 220 | 24.4 | 7.49 | 0.135 |
| Q6UWP8 | SBSN | 15.3 | 2 | 3 | 2 | 590 | 60.5 | 7.01 | 0.236 |
| Q9Y275 | TNFSF13B | 4.6 | 1 | 2 | 1 | 285 | 31.2 | 6.25 | 0.470 |
| Q13790 | APOF | 7.7 | 2 | 8 | 2 | 326 | 35.4 | 5.64 | 0.250 |
| P32004 | L1CAM | 1.0 | 1 | 1 | 1 | 1257 | 139.9 | 6.24 | 0.078 |

| | | | | | | | | | |
|---|---|---|---|---|---|---|---|---|---|
| P08254 | MMP3 | 2.5 | 1 | 1 | 1 | 477 | 53.9 | 6.16 | 0.241 |
| P08253 | MMP2 | 55.0 | 30 | 98 | 30 | 660 | 73.8 | 5.47 | 0.181 |
| Q9Y5Y7 | LYVE1 | 15.2 | 5 | 14 | 5 | 322 | 35.2 | 8.28 | 0.138 |
| P60709 | ACTB | 61.1 | 17 | 104 | 1 | 375 | 41.7 | 5.48 | 0.146 |
| P46939 | UTRN | 0.4 | 1 | 1 | 1 | 3433 | 394.2 | 5.33 | 0.388 |
| P11226 | MBL2 | 31.5 | 6 | 20 | 6 | 248 | 26.1 | 5.49 | 0.052 |
| A0A087WW87 | IGKV2-40 | 10.7 | 1 | 5 | 1 | 121 | 13.3 | 4.61 | 0.322 |
| P45877 | PPIC | 6.1 | 1 | 2 | 1 | 212 | 22.7 | 8.4 | 0.115 |
| P98066 | TNFAIP6 | 7.2 | 1 | 1 | 1 | 277 | 31.2 | 6.79 | 0.230 |
| P02649 | APOE | 74.4 | 25 | 158 | 25 | 317 | 36.1 | 5.73 | 0.142 |
| P07585 | DCN | 10.6 | 4 | 10 | 4 | 359 | 39.7 | 8.54 | 0.186 |
| Q86TH1 | ADAMTSL2 | 4.7 | 2 | 2 | 2 | 951 | 104.6 | 6.42 | 0.083 |
| P04114 | APOB | 59.9 | 259 | 2604 | 259 | 4563 | 515.3 | 7.05 | 0.202 |
| O14791 | APOL1 | 19.1 | 8 | 15 | 8 | 398 | 43.9 | 5.81 | 0.239 |
| A0A075B6I9 | IGLV7-46 | 7.7 | 1 | 1 | 1 | 117 | 12.5 | 7.2 | 0.396 |
| Q7LFX5 | CHST15 | 2.3 | 1 | 1 | 1 | 561 | 64.9 | 8.29 | 0.171 |
| P04278 | SHBG | 46.8 | 14 | 74 | 14 | 402 | 43.8 | 6.71 | 0.119 |
| Q9UEW3 | MARCO | 8.1 | 4 | 5 | 4 | 520 | 52.6 | 8.78 | 0.254 |
| Q13093 | PLA2G7 | 16.1 | 6 | 6 | 6 | 441 | 50.0 | 7.56 | 0.226 |
| Q8N9V7 | TOPAZ1 | 1.2 | 1 | 1 | 1 | 1692 | 190.8 | 7.87 | 0.163 |
| P35542 | SAA4 | 27.7 | 4 | 15 | 4 | 130 | 14.7 | 9.07 | 0.186 |
| P02741 | CRP | 23.2 | 5 | 18 | 5 | 224 | 25.0 | 5.63 | 0.159 |
| P16233 | PNLIP | 9.0 | 3 | 3 | 3 | 465 | 51.1 | 6.73 | 0.376 |
| Q6WN34 | CHRDL2 | 1.6 | 1 | 1 | 1 | 429 | 47.5 | 7.88 | 0.053 |
| P02654 | APOC1 | 34.9 | 5 | 18 | 5 | 83 | 9.3 | 8.47 | 0.318 |
| P02656 | APOC3 | 34.3 | 3 | 21 | 3 | 99 | 10.8 | 5.41 | 0.331 |
| P10645 | CHGA | 7.4 | 2 | 3 | 2 | 457 | 50.7 | 4.6 | 0.177 |
| P02655 | APOC2 | 49.5 | 4 | 15 | 4 | 101 | 11.3 | 4.72 | 0.248 |
| P48304 | REG1B | 21.1 | 3 | 11 | 2 | 166 | 18.7 | 5.95 | 0.104 |
